# Repurposing endogenous immune pathways to tailor and control chimeric antigen receptor T cell functionality

**DOI:** 10.1038/s41467-019-13088-3

**Published:** 2019-11-13

**Authors:** Mohit Sachdeva, Brian W. Busser, Sonal Temburni, Billal Jahangiri, Anne-Sophie Gautron, Alan Maréchal, Alexandre Juillerat, Alan Williams, Stéphane Depil, Philippe Duchateau, Laurent Poirot, Julien Valton

**Affiliations:** 1 Cellectis, Inc., 430 East 29th Street, New York, NY 10016 USA; 2 Cellectis, 8 rue de la Croix Jarry, 75013 Paris, France

**Keywords:** Cytokines, Immunotherapy, Translational immunology, Cancer

## Abstract

Endowing chimeric antigen receptor (CAR) T cells with additional potent functionalities holds strong potential for improving their antitumor activity. However, because potency could be deleterious without control, these additional features need to be tightly regulated. Immune pathways offer a wide array of tightly regulated genes that can be repurposed to express potent functionalities in a highly controlled manner. Here, we explore this concept by repurposing TCR, CD25 and PD1, three major players of the T cell activation pathway. We insert the CAR into the TCRα gene (TRAC_CAR_), and IL-12P70 into either IL2Rα or PDCD1 genes. This process results in transient, antigen concentration-dependent IL-12P70 secretion, increases TRAC_CAR_ T cell cytotoxicity and extends survival of tumor-bearing mice. This gene network repurposing strategy can be extended to other cellular pathways, thus paving the way for generating smart CAR T cells able to integrate biological inputs and to translate them into therapeutic outputs in a highly regulated manner.

## Abstract (web summary)

Engineered T cells work as living therapeutics, but are prone to hyperreactivity and exhaustion. Here the authors improve CAR T cell antitumor responses by simultaneously targeting a CAR to TCR locus and IL-12 to PD1 locus, placing the transgenes under a naturally regulated transcriptional network while disrupting unwanted signals.

## Introduction

Chimeric Antigen Receptor (CAR) T cell and T cell therapies hold great promise for treating a wide range of malignancies. However, these therapies face multiple challenges, including tumor accessibility, tumor-dependent inhibitory signals, and a microenvironment that is hostile to T cells. To overcome these obstacles, CAR T cells have been engineered to remove inhibitory receptors^1–3^, to express costimulatory molecules^3^ and chemokine receptors^4,5^, or to secrete immunostimulatory factors such as checkpoint inhibitors^6^ or cytokines^7^. In particular, cytokine secretion by so called Armored or Truck CAR T cells^7,8^ is a highly promising strategy for improving CAR T cell function. When integrated into the T cell genome via lentiviral or gamma retroviral vector transduction, recombinant IL-12P70^9–12^, IL-15^13,14^, and IL-18^15–17^ have been reported to increase CAR T cell antitumor activity, to prevent CAR T cell anergy, to remodel the tumor microenvironment, and to recruit the innate immune cells that are necessary for the antitumor response^9^. However, as these immunostimulatory agents are highly potent, they may have side effects^18^. When secreted by CAR T cells or by Tumor Infiltrating Lymphocytes (TILs), they can generate adverse preclinical^12,19^ and clinical^20^ events, given the suboptimal control of their secretion and the high quantity of factor released in the circulation^20,21^. Thus, new engineering strategies that enable tight control of the production, secretion, or presentation of exogenous proteins of interest by engineered T cells will be valuable in developing the next generation of T cell-based therapies.

Immune pathways, which evolved to support the survival of the host in response to infectious agents^22^, offer a wide array of tightly regulated genes that can be repurposed to secrete a protein of interest, such as a cytokine, in a conditional and highly controlled manner. For example, the activation of T cells via T cell receptor (TCR) engagement triggers a multistep signaling process that leads to the activation of transcription factors, including Nuclear Factor of Activated T cells (NFAT), which orchestrate a precise ballet of gene expression and subsequent protein translation^23^. Some of these genes, including IL-2, CD69, and IL2Rα, are rapidly induced to promote T cell proliferation, differentiation, and activation, whereas others, including PDCD1, LAG3, and FASLG, are induced later to serve as a rheostat for the immune response^24,25^. Thanks to transcriptional and post-translational regulation, the coordinated induction of these genes follows precise kinetics and quantitative patterns that fine-tune the functional outputs of T cells. Customizing these functional outputs by repurposing endogenous gene expression therefore represents an attractive means for generating smart CAR T cells that are able to sense and react to their environment in a tailored, highly regulated, and antigen-specific manner.

We explored this concept by repurposing TCR, CD25, and PD1, three major players of the T cell activation pathway, to enable CAR T cells to secrete the pro-inflammatory cytokine IL-12P70 in a tumor cell-dependent manner. Based on the seminal work established in^26^, we use the TALEN technology with recombinant adeno-associated virus 6 (AAV6) repair vectors to place CAR and IL-12P70 expression under the control of TCR (TRAC_CAR_)^26^ and either IL2Rα or PDCD1 regulatory elements, respectively. This multiplex gene repurposing process leads to the expression of a CAR and to conditional secretion of IL-12P70. The secretion of IL-12P70 is transient, dependent on tumor engagement, and follows the tightly regulated patterns of CD25 and PD1 expression that are observed upon T cell activation. IL-12P70 promotes the accumulation of TRAC_CAR_ T cells and markedly improves their antitumor activity in vitro and in vivo. Furthermore, the targeted integration of IL-12P70 at the PDCD1 locus concomitantly inactivate PD1, one of the major checkpoint of T cell function.

## Results

### TCR pathway repurposing via multiplex gene editing

**Fig. 1:**
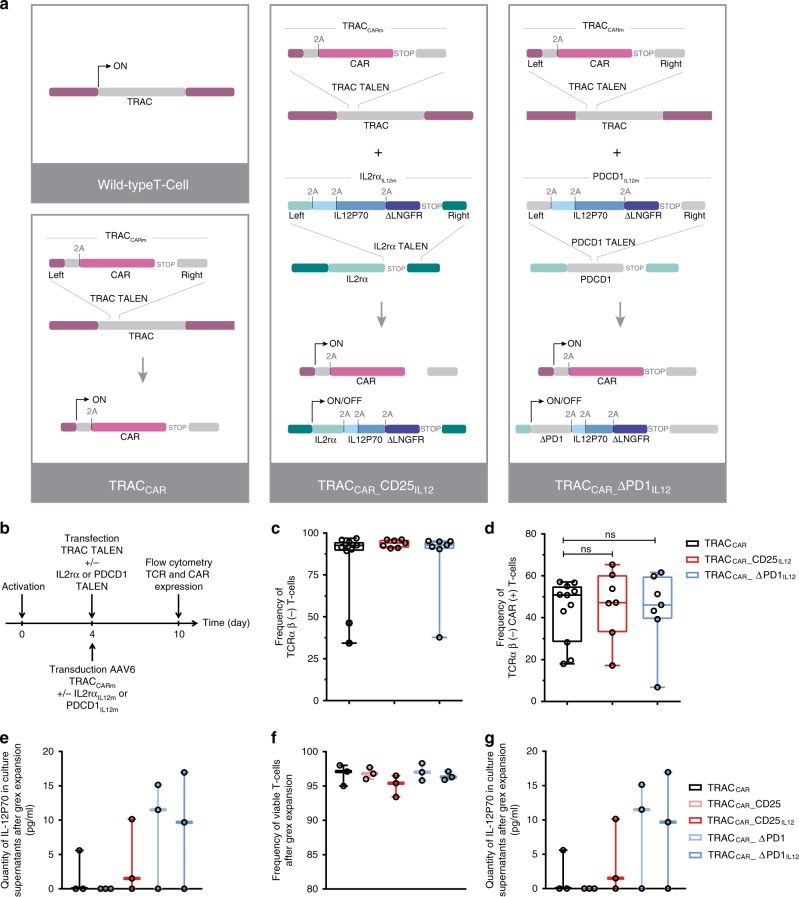
Repurposing the TCR pathway via TALEN and adeno-associated virus (AAV6) treatments. **a** Schematic showing the strategies used to repurpose TRAC along with the IL2rα or PDCD1 loci to generate TRAC_CAR_, TRAC_CAR__CD25_IL12_, and TRAC_CAR__ΔPD1_IL12_ T cells from wild-type T cells. The genetic elements composing the TRAC and IL-12P70 matrices (TRAC_CARm_, IL2rα_IL12m_ and PDCD1_IL12m_) are illustrated. TRAC_CARm_ was designed to express a CAR architecture via the TRAC locus, and IL2rα_IL12m_ and PDCD1_IL12m_ were designed to express IL-12P70 and ΔLNGFR via the IL2rα and PDCD1 loci. The three matrices keep the reading frame of the targeted genes, and they contain 2A self-cleaving elements upstream of each expression cassette and 300-bp homology arms that are specific for each locus. The matrix insertion locations were chosen to inactivate TCR and PD1 (expression of truncated/inactive form after insertion) and to preserve CD25 expression. **b** Experimental design for multiplex repurposing of TRAC, IL2rα or PDCD1 and analysis of the resulting engineered TRAC_CAR_ T cells. **c** and **d** Frequency of TCRαβ (−) T cells and of TCRαβ (−) CAR (+) T cell observed by flow cytometry, respectively, 6 days post TALEN mRNA electroporation and AAV6 transduction. Each point represents one experiment performed with a given donor. *n* = 11 for TRAC_CAR_, *n* = 7 for TRAC_CAR__CD25_IL12_ and *n* = 7 for TRAC_CAR__ΔPD1_IL12_ T cells. One-way ANOVA was used in panel **d** for statistical analysis (ns not significant). **e**–**g** Summary of engineered TRAC_CAR_ T cell production. TRAC_CAR_ T cells generated from 3 independent donors were analyzed at the end of their production. **e**, **f**, and **g** show the number of TRAC_CAR_ T cells obtained at the end of a GREX10 culture, the frequency of viable cells, and the quantity of IL-12P70 detected in the culture supernatants (Lower limit of IL12P70 detection 9.8 pg/mL), respectively. Each point represents one experiment performed with a given donor. *n* = 3 for each engineered TRAC_CAR_ T cells batch. On each box plot, the central mark indicates the median, the bottom and top edges of the box indicate the interquartile range (IQR) and the whiskers represent the maximum and minimum data points. Source data are provided as a Source Data file

Targeted insertion via homology-directed repair is possible in primary T cells and hematopoietic stem and progenitor cells. Techniques for such insertion include AAV6 or DNA repair matrices along with site-specific engineered nucleases such as TALEN, megaTAL, meganucleases, Zinc Finger nucleases and CRISPR-CAS9^26–35^. Here, we extend this approach pioneered by^26^ to simultaneously engineer CAR and IL-12P70 expression under the control of TRAC and either IL2rα or PDCD1 regulatory elements (Fig. 1a). To do so, we generated three different AAV6 promoter-less repair matrices. The first matrix, TRAC_CARm_, was designed to integrate an anti-CD22 CAR expression cassette at the TRAC locus^26,36^. This CAR architecture contains integrated purification and safeguard domains that allow purification and Rituximab-dependent depletion of TRAC_CAR_ T cells (Supplementary Fig. 1a)^36^. The second and third matrices, IL2rα_IL12m_ and PDCD1_IL12m_, were designed to integrate the IL-12P70 heterodimer (formed by IL-12A p35 and IL-12B p40 subunits) along with a truncated low-affinity nerve growth factor receptor (ΔLNGFR) surface marker^37^ at the IL2rα and PDCD1 loci, respectively. These three matrices use the reading frame of the targeted genes and contain 2A self-cleaving elements upstream of each expression cassette and 300 bp homology arms specific for TRAC, IL2rα, or PDCD1 loci. The insertion sites for the matrices allow functional inactivation of TCR and PD1 (leading to a truncated and inactive form) and preserve the expression of CD25, an important component of the IL-2 receptor^38,39^ (Fig. 1a).

To demonstrate that CAR and IL-12P70 expression can be placed under the control of the TRAC and IL2rα or PDCD1 regulatory elements, respectively, we simultaneously transduced T cells with TRAC_CARm_ and either IL2rα_IL12m_ or PDCD1_IL12m_ after TALEN treatment (Fig. 1b) to generate TRAC_CAR__CD25_IL12_, or TRAC_CAR__ΔPD1_IL12_ T cells, respectively (Fig. 1a). Reference controls consisted in treating only with TRAC TALEN and TRAC_CARm_ repair template (named TRAC_CAR_ cells), or by simply omitting the IL-12 repair matrix while maintaining double TALEN treatment and CAR transduction (TRAC_CAR__CD25 and TRAC_CAR__ΔPD1 T cells), were generated to account for the effect of IL2Rα or PDCD1 TALEN independently of IL-12. We observed efficient TCR knockout (>90% TCRαβ (−) T cells, Fig. 1c) and targeted expression of CAR at the TRAC locus (up to 65% of CAR (+) TCRαβ (−) T cells, Fig. 1d) 6 days after treatment. Similar results were obtained under the three different conditions, indicating that the presence of multiple TALEN constructs and the IL-12P70 repair matrix do not disturb the targeted CAR insertion at the TRAC locus. This engineering process did not affect the viability or the proliferation capacity of TRAC_CAR_ T cells (Fig. 1e, f) and led to low levels of IL-12P70 secretion in the culture media after 15 days of expansion (lower level of detection was determined at 9.8 pg/mL). Furthermore, it did not affect our ability to purify and efficiently deplete TRAC_CAR_ T cells with the FDA-approved drug Rituximab (RTX), as demonstrated previously using a lentiviral-based vectorization approach (Supplementary Figs. 1 and 2^36^).

### Specificity of TCR pathway repurposing

**Fig. 2:**
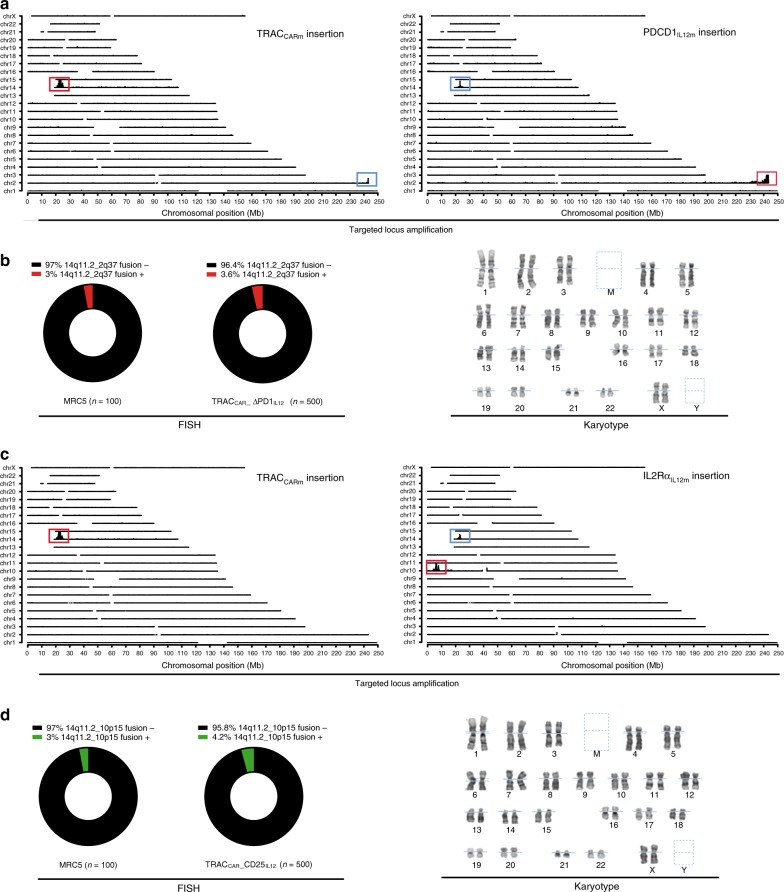
Cytogenetic and genomic characterization of engineered TRAC_CAR_ T cells. **a** Targeted locus amplification (TLA) analysis of TRAC_CAR__ΔPD1_IL12_ cells using TRAC_CARm_ specific primers (left panel) or PDCD1_IL12m_ specific primers (right panel). Specific targeted integration coverages and potential concatemere or homology-independent integrations are indicated by red and blue boxes, respectively. **b**, left panel Frequency of translocation events between TRAC and PDCD1 loci detected by FISH in MRC5 standard cell line (*n* = 100 metaphases analyzed) and in TRAC_CAR__ΔPD1_IL12_ (*n* = 500 metaphases analyzed). Cells harboring or not the expected gene fusion named 14q11.2_2q37 are illustrated in red or black, respectively and the number of metaphase analyzed for each sample are indicated. **b**, right panel Representative example of TRAC_CAR__ΔPD1_IL12_ cells karyotype showing normal ploidy level and no sign of genetic alteraction of TRAC and PDCD1 chromosomal loci. **c** Targeted locus amplification analysis of TRAC_CAR__CD25_IL12_ cells using TRAC_CARm_ specific primers (left panel) or IL2rα_IL12m_ specific primers (right panel). Specific targeted integration coverages and potential concatemere integrations are indicated by red and blue boxes, respectively. **d**, left panel Frequency of translocation events between TRAC and IL2rα loci detected by FISH in MRC5 standard cell line (*n* = 100 metaphases analyzed) and in TRAC_CAR__CD25_IL12_ (*n* = 500 metaphases analyzed). Cells harboring or not the expected gene fusion named 14q11.2/10p15.1 are indicated in red and black, respectively and the number of metaphase analyzed for each sample are indicated. Chi square was used to calculate the statistical significance of FISH analysis. **d**, right panel Representative example of TRAC_CAR__CD25_IL12_ karyotype showing normal ploidy and no sign of genetic alteration of IL2rα and TRAC chromosomal loci. The TLA and FISH plots and Karyotype figures represents 1 experiment performed with 1 donor. Source data are provided as a Source Data file

Targeted insertion of the three matrices was confirmed using Targeted Locus Amplification (TLA), a method enabling genome wide, unbiased mapping of transgene insertion^40^ (Fig. 2). As expected, TRAC_CARm_, IL2rα_IL12m_ and PDCD1_IL12m_ were specifically inserted into chromosome 14, 10, or 2 respectively (Fig. 2a, c). The majority of transgenes were inserted in frame with the intended open reading frame. We also found low coverage of TRAC_CARm_ on chromosome 10 or 2 (PDCD1 and IL2rα loci) and of IL2rα_IL12m_ or PDCD1_IL12m_ on chromosome 14 (TRAC Locus). The molecular signature of these marginal events suggests co-integration of matrices concatemeres at TRAC, IL2rα, and PDCD1 loci in a few cells, although we cannot exclude homology-independent trapping of matrices at the three loci.

Because simultaneous TALEN treatment could lead to unwanted genetic adverse events including translocation between two targeted loci^41,42^, we characterized cytogenic properties of engineered TRAC_CAR__CD25_IL12_ and TRAC_CAR__ΔPD1_IL12_ T cells thoroughly by karyotyping and Fluorescence-In-Situ-Hybridization (FISH). Karyotyping and G-banding pattern results were consistent with human diploid samples devoid of gross structural abnormalities or clonal chromosomal aberrations (Fig. 2b, d). Consistently, FISH analysis did not show significant difference (*p* = 0.766, Chi square) between the frequency of TRAC/PDCD1 or TRAC/IL2Rα gene fusion obtained in engineered T cells and in MRC5, a human fibroblast cell line considered a reference diploid cell line and commonly used as a negative control for gene fusion detection (Fig. 2b, d). Although the low sensitivity of this test does not rule out translocation (sensitivity of FISH analysis ~ 1–4%), it indicated that they were not markedly enriched over the standard expansion process used to produce TRAC_CAR_ T cells. Further work will be needed to thoroughly address this point before moving toward clinical applications.

### Disruptive and non disruptive targeted insertion of IL-12P70

**Fig. 3:**
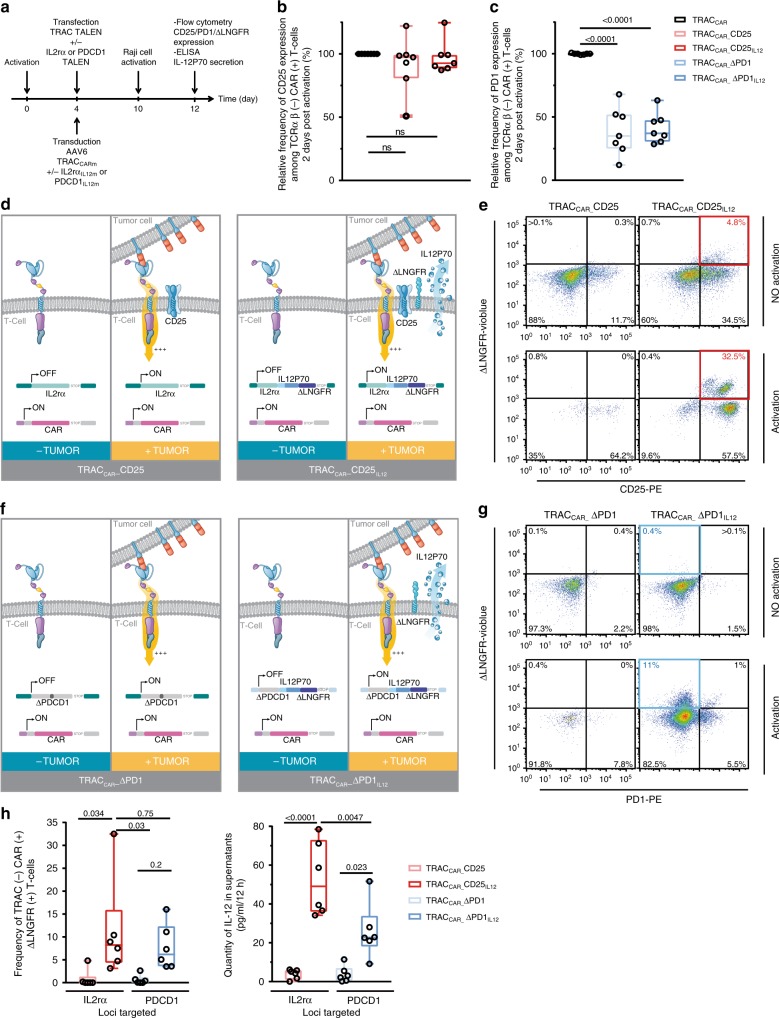
Efficient translation of tumor cell engagement by TRAC_CAR_ T cell into IL-12P70 secretion in vitro. **a** Schematic showing the experimental design used to determine the levels of CD25 and PD1 expression and IL-12P70 secretion by tumor cell-activated TRAC_CAR_ T cells. **b** and **c** Frequency of CD25 and PD1 expression detected among TCRαβ (−) and CAR (+) T cells, respectively. Each point represents one experiment performed with a given donor (*n* = 7). One-way ANOVA was used in panel **3b** and **3c** for statistical analysis and *p*-value are indicated. **d** Schematic showing the expected outcome of IL-12P70 secretion upon tumor cell engagement by TRAC_CAR__CD25 or TRAC_CAR__CD25_IL12_ T cells. **e** Flow cytometry plots showing ΔLNGFR surface expression 48 h after tumor cell-dependent activation of TRAC_CAR__CD25 and TRAC_CAR__CD25_IL12_ T cells. The flow plot was obtained using engineered TRAC_CAR_ T cell batches harboring the highest IL-12P70 targeted integration. The red box indicates the proportion of ∆LNGFR expressed by successfully engineered TRAC_CAR_ T cells. Gating strategy is illustrated in Supplementary Fig. 4. **f** Schematic showing IL-12P70 secretion upon tumor cell engagement by TRAC_CAR__ΔPD1 or TRAC_CAR__ΔPD1_IL12_ T cells. **g** Flow cytometry plots showing the level of ΔLNGFR expression, 48 h after tumor cell-dependent activation of TRAC_CAR__CD25 and TRAC_CAR__CD25_IL12_ T cells. The solid blue squares indicate the frequency of ∆LNGFR expression by successfully engineered TRAC_CAR_ T cells. Gating strategy is illustrated in Supplementary Fig. 4. **h** Frequency of ∆LNGFR expression (left panel) and quantity of IL-12P70 secreted (right panel) by TRAC_CAR_ T cells engineered at the IL2rα and PDCD1 loci, 48 h after tumor cell-dependent activation of the TRAC_CAR_ T cells. Each point represents one experiment performed with a given donor (*n* = 6) and the threshold of IL-12P70 detection is 5 pg/mL. On each box plot, the central mark indicates the median, the bottom and top edges of the box indicate the interquartile range (IQR) and the whiskers represent the maximum and minimum data point. One-way ANOVA was used in panel **3h** for statistical analysis and *p*-value are indicated. Source data are provided as a Source Data file

After demonstrating targeted insertion of the three matrices, we verified that the delivery of PDCD1-specific and IL2rα-specific TALEN disrupted PD1 and spared CD25 surface presentation in the presence or absence of appropriate IL-12P70 repair matrices. After activation of engineered TRAC_CAR_ T cells with RAJI cells (E/T = 1) and T cell analysis by flow cytometry to determine CD25 and PD1 expression (Fig. 3a), we observed similar frequencies of CD25 expression among the three different conditions (Fig. 3b). This indicated that TALEN-mediated processing of the 3’UTR of IL2rα gene (Supplementary Fig. 3a) did not impair CD25 surface expression. In contrast, PD1 expression was significantly impaired, as illustrated by the difference between the surface expression of PD1 in the PDCD1 TALEN-treated TRAC_CAR_ T cells (TRAC_CAR__ΔPD1 and TRAC_CAR__ΔPD1_IL12_ T cells) and that of the TRAC_CAR_ T cell control (Fig. 3c, one way Anova, *p* < 0.0001 for both comparisons). Consistently, high throughput DNA sequencing showed that PDCD1 TALEN treatment promoted 66% of indels at the PDCD1 locus (Supplementary Fig. 3a). Because of its potential for downstream clinical application, we further characterized the specificity of TRAC/PDCD1 TALEN co-treatment using an Oligo capture assay derived from ref. ^43^ and high throughput DNA sequencing (Supplementary Fig. 3b). The potential offsite candidates identified in T cells treated with TRAC and PD1 TALEN showed similar frequency of insertion and deletions (Indels) than the ones detected in mock-treated T cells. Thus, TRAC/PDCD1 TALEN co-treatment did not lead to offsite targeting above the natural mutation rate observed in primary T cells.

### Translating tumor cell engagement into secretion of IL-12P70

We indirectly determined the efficiency of IL-12P70 matrix insertion at the IL2rα or PDCD1 loci by assessing the expression of ΔLNGFR at the surface of TRAC_CAR_ T cells and characterizing IL-12P70 secretion upon tumor cell engagement. For this purpose, we activated TRAC_CAR_ T cells with RAJI cells as described above (Fig. 3a) and then analyzed T cells using flow cytometry. We observed an increase in ΔLNGFR expression in TRAC_CAR_ T cells engineered to express IL-12P70 under the control of IL2rα regulatory elements (Fig. 3e, red square). Consistently, ΔLNGFR was only detected in CD25 (+) TRAC_CAR_ T cells, suggesting an efficient targeted insertion of the IL-12P70 matrix at the IL2rα locus. Similar results were obtained with TRAC_CAR_ T cells engineered to express IL-12P70 under the control of PDCD1 regulatory elements, in which we also observed an increase of ΔLNGFR upon tumor cell engagement (Fig. 3g, blue square). However, in contrast to the IL2rα locus, the majority of ΔLNGFR-positive cells were PD1-negative, as expected for a matrix designed to interrupt the PDCD1 coding sequence. A significant increase of ΔLNGFR expression by TRAC_CAR__CD25_IL12_ T cells compared to TRAC_CAR__CD25 T cells (*p* = 0.034, one-way ANOVA) and a marked increase of ΔLNGFR expression by TRAC_CAR__ΔPD1_IL12_ T cells compared to TRAC_CAR__ΔPD1 T cells was observed for multiple T cell donors engineered to target the IL2rα and PDCD1 loci (Fig. 3h).

To determine whether there was a correlation between the tumor cell-dependent expression of ΔLNGFR by TRAC_CAR_ T cells and IL-12P70 secretion, we characterized the supernatants recovered after two days of TRAC_CAR_ T cell/tumor cell co-culture through ELISA quantification (Fig. 3a and Fig. 3h right panel). TRAC_CAR_ T cells secreted medians of 23 or 49 pg of IL-12P70/mL/12 h when the IL-12P70 matrix was inserted at the PDCD1 locus or the IL2rα locus, respectively. In contrast, TRAC_CAR_ T cells lacking IL-12P70m secreted significantly less IL-12P70 (one way Anova, *p* = 0.023 and *p* < 0.0001 for PDCD1 and the IL2rα locus, respectively) showing levels of secreted IL-12P70 lying within the threshold of detection (5 pg/mL). These results indicate that IL2rα or PDCD1 can be rewired to secrete IL-12P70 in a tumor cell-dependent manner.

### Secretion of IL-12P70 by TRAC_CAR_ T cell is tighly regulated

**Fig. 4:**
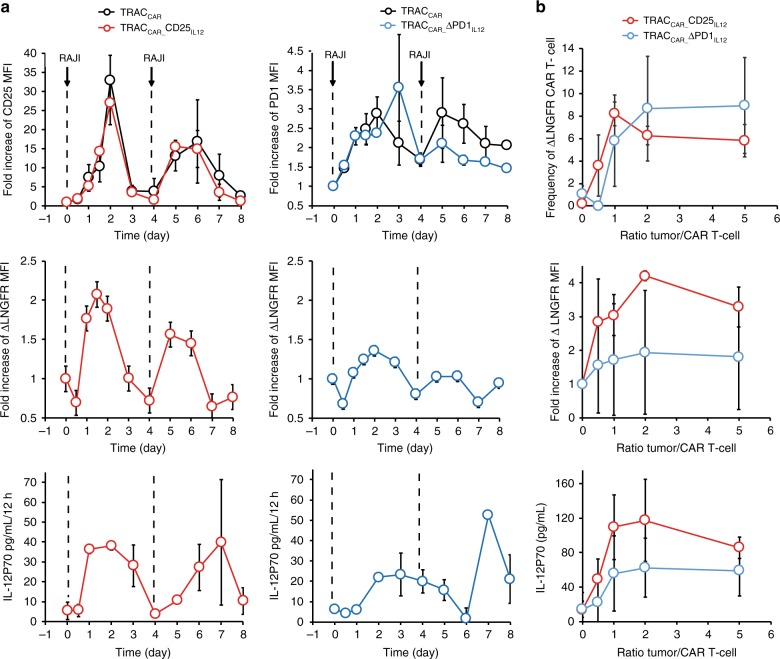
The secretion of IL-12P70 by TRAC_CAR_ T cells is regulated by tumor cell engagement in vitro. **a** Kinetics of CD25 and PD1 expression (top), ΔLNGFR expression (middle), and IL-12P70 secretion (bottom panel) in purified TRAC_CAR_ T cells after two consecutive tumor cells challenges. TRAC_CAR_ T cells were activated by RAJI cells at day 0 and day 4 and analyzed by flow cytometry for 8 days. IL-12P70 secreted in cell culture supernatants was quantified by ELISA and plotted as a function of time. The arrows indicate the times of tumor cell addition. The data shown represent the average +/− standard deviation of two experiments performed with cells from two different donors. **b** Changes in ΔLNGFR reporter expression and IL-12P70 secretion as a function of tumor cell concentration. Engineered TRAC_CAR_ T cells were activated by different quantities of RAJI cells for 48 h. Supernatants were used to quantify IL-12P70 via ELISA assays and cells were analyzed by flow cytometry to evaluate the frequency of ΔLNGFR+ cells, as well as its mean fluorescence intensity. Values are plotted as a function of the tumor cell to TRAC_CAR_ T cell ratio. The data shown are the average +/− standard deviation of three experiments performed with cells from three different donors. Source data are provided as a Source Data file

To explore the temporal regulation of IL-12P70 in response to tumor cell engagement, we characterized the kinetics of CD25, PD1, and ΔLNGFR expression upon repeated stimulations with tumor cells. To this end, we cultured TRAC_CAR_ T cells with tumor cells on days 0 and 4 and monitored surface protein expression by flow cytometry (Fig. 4). We observed a biphasic pattern of PD1 and CD25 expression after tumor cell engagement by TRAC_CAR_ T cells (Fig. 4a, top panels). This trend, reproduced after the second tumor cell engagement, indicates that the expression of both proteins is tightly upregulated and downregulated upon CAR activation. This regulation was also observed for ΔLNGFR expression and IL-12P70 secretion, demonstrating that the ΔLNGFR and IL-12P70 transgenes benefited from the IL2rα and PDCD1 endogenous regulatory elements (Fig. 4a). Notably, ΔLNGFR and IL-12P70 were induced faster than CD25 after the first tumor cell engagement, suggesting that there are differences in the trafficking or cell surface multimerization kinetics between these proteins. In addition, while the downregulation of CD25 and PD1 led to an almost complete shutdown of their expression, this phenomenon showed subtle kinetic differences that paralleled ΔLNGFR expression and IL-12P70 secretion levels. Indeed, PD1 expression was still detectable at day 3, when CD25 expression reached its basal level. This difference was also observed for the corresponding ΔLNGFR surrogate and for IL-12P70, found to be secreted over a longer time by TRAC_CAR__ΔPD1_IL12m_ than by TRAC_CAR__CD25_IL12m_. Similar results were obtained when IL-15/IL-15rα was substituted for IL-12P70, demonstrating the robustness and transposability of our approach to express proteins of therapeutic interest^44,45^ in a tumor cell-dependent manner (Supplementary Fig. 4).

We benchmarked our repurposing strategy to the engineering approach developed earlier to reexpress IL-12P70^20,21,46,47^ in a tumor cell-dependent manner. To do so, we first assessed the level of IL-12P70 secreted by T cell transduced with increasing amounts of rLV particles encoding NFAT IL-12P70, a construct previously designed to express an IL-12P70 under the control of 6xNFAT promoter^20,21^. Transduced cells obtained from one donor were co-cultivated with and without PMA ionomycin to determine the basal and activation-dependent levels of IL-12P70 secretion. Consistent with former studies^21,48^, IL-12P70 was efficiently secreted upon activation, but also detected in the absence of activation indicating a suboptimal control of secretion. To confirm this aspect, we compared the levels of IL12P70 secretion by TRAC_CAR__ΔPD1_IL12_ and by CAR T cell transduced with low MOI of NFAT IL-12P70 rLV particle (CAR_NFAT_IL12_, MOI = 0.25) before, during, and after tumor cell engagement (Supplementary Fig. 6d). Our results showed that both engineering strategies enable tumor-cell-dependent secretion of IL-12P70. However, CAR_NFAT_IL12_ displayed basal leakiness, as illustrated by the presence of substantial levels of IL-12P70 before and after 8 days of tumor cell challenge (Supplementary Fig. 6d). This pattern contrasted with the one obtained from TRAC_CAR__ΔPD1 IL12 cells, consistently showing low secretion of IL-12P70 before and after 8 days of tumor cell challenge.

To characterize the control of gene expression after tumor cell engagement further, we assessed if an increased tumor cell concentration correlated with the level of IL-12P70 secretion by TRAC_CAR_ T cells. To do so, we incubated TRAC_CAR_ T cells in the presence of increasing concentration of tumor cells for two days and measured the levels of secreted IL-12P70 (Fig. 4b). The extent of IL-12P70 secretion increased as a function of tumor cell concentration and reached a plateau at a tumor-to-TRAC_CAR_ T cell ratio of 1-to-1 (Fig. 4b). Interestingly, the repurposed IL2rα locus elicited a higher maximum level of IL-12P70 secretion than the PDCD1 locus. This correlated with the significant differences observed between the quantity of IL-12P70 secreted by PDCD1 and IL2rα loci (Fig. 3h right, *p* ≤ 0.023, one-way ANOVA) and suggests that the level of therapeutic product secretion could be controlled by careful selection of a locus to repurpose.

### Tumor-dependant and local delivery of IL-12P70 in vivo

**Fig. 5:**
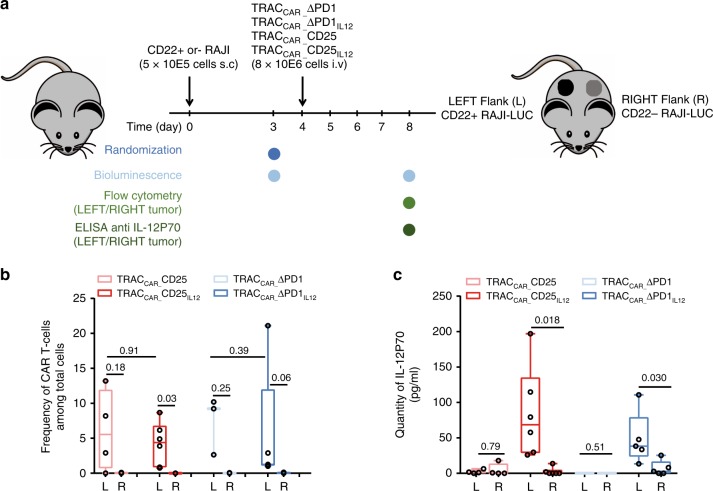
In vivo local and tumor-dependent delivery of IL-12P70 by TRAC_CAR_ T cells. **a** Schematic showing the experimental design to study the tumor-dependent secretion of IL-12P70 by TRAC_CAR_ T cells in NSG mice (BLI: bioluminescence). CD22(+) and CD22(−) RAJI tumor cells expressing firefly luciferase were transferred subcutaneously into the left (L) and right (R) flanks of NSG mice, respectively, and allowed to engraft for 4 days before TRAC_CAR_ T cell transfer. Four days after TRAC_CAR_ T cells transfer, tumors and T cells were recovered, dissociated, and analyzed via flow cytometry and ELISA. **b** Frequency of TRAC_CAR_ T cells detected by flow cytometry at the left (L) and right (R) flanks of NSG mice. A non parametric paired Mann-Whitney test was used for statistical analysis of the data (*p*-value are indicated). **c** Quantity of IL-12P70 detected in the left (L) and right (R) flanks of NSG mice. Each point documented in **b** and **c** represents the result obtained in each mice (*n* = 4 for TRAC_CAR__CD25, *n* = 6 for TRAC_CAR__CD25_IL12_, *n* = 3 for TRAC_CAR__ΔPD1, *n* = 5 for TRAC_CAR__ΔPD1_IL12_). On each box plot, the central mark indicates the median, the bottom and top edges of the box indicate the interquartile range (IQR) and the whiskers represent the maximum and minimum data point. A paired Wilcoxon-Mann-Whitney non-parametric test was used for statistical analysis of the IL-12P70 quantities obtained at the right and left flanks of the same animal (p-value are indicated). Source data are provided as a Source Data file

As the IL2rα and PDCD1 loci can be efficiently repurposed to secrete IL-12P70 in a controlled and tumor-dependent manner in vitro, we tested whether the IL-12P70 secreting TRAC_CAR_ T cells could transiently and locally deliver IL-12P70 to an engrafted tumor in a xenogenic mouse model. For that purpose, CD22(+) and CD22(−) RAJI tumor cells expressing firefly luciferase were transplanted subcutaneously into the left and right flanks of NSG mice, respectively (Fig. 5a). Mice were then randomized on the basis of their luminescence signal three days after transplantation and injected intravenously with TRAC_CAR__CD25, TRAC_CAR__CD25_IL12_, TRAC_CAR__ΔPD1, or TRAC_CAR__ΔPD1_IL12_ T cells. Four days after TRAC_CAR_ T cell transfer, the tumors were analyzed by flow cytometry and ELISA. TRAC_CAR_ T cells were robustly detected at the CD22(+) tumor site (left flank) but were almost absent from the CD22(-) tumor site (right flank, Fig. 5b, left graph), indicating that TRAC_CAR_ T cells preferentially accumulate at their intended tumor-specific site. The frequencies of TRAC_CAR_ T cells detected at the CD22(+) tumor sites were similar in all groups regardless of IL-12P70 status (Fig. 5b, left graph), suggesting that genetic engineering does not affect the trafficking or early accumulation of T cells. IL-12P70 secretion was significantly lower in the CD22(−) right tumor site than in the CD22(+) left tumor site (Fig. 5c, Wilcoxon-Mann-Whitney test, *p* = 0.018 and 0.03 for TRAC_CAR__CD25_IL12_ and TRAC_CAR__ΔPD1_IL12_, respectively), suggesting that IL-12P70 can be conditionally and locally delivered to a specific tumor site in vivo by TRAC_CAR_ T cells.

### Secretion of IL-12P70 improves TRAC_CAR_ T cells activity

**Fig. 6:**
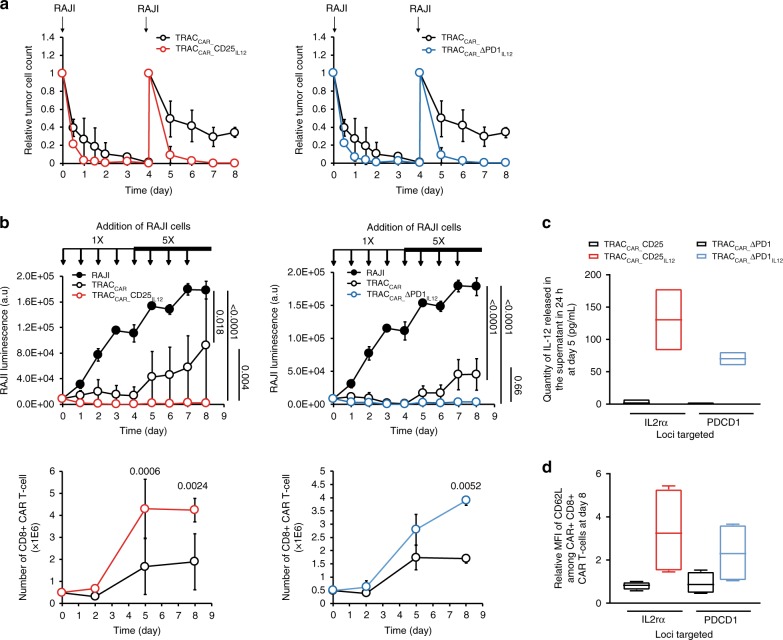
Conditional secretion of IL-12P70 increases TRAC_CAR_ T cells antitumor activity in vitro. **a** Kinetics of tumor cell clearance by purified TRAC_CAR_ T cells and TRAC_CAR_ T cells secreting IL-12P70. TRAC_CAR_, TRAC_CAR__CD25_IL12_ T cells and TRAC_CAR__ΔPD1_IL12_ T cells were challenged with 1 equivalent of RAJI cells at days 0 and 4 and cultivated for 8 days. Kinetics of tumor cell disappearance was analyzed by integrating flow cytometry data, using CD19 antibody specific for RAJI cells, and total number of cells mixture. The relative tumor cell count was computed as the ratio of remaining tumor cells at a given time to the total number of tumor cells added. **b** Long-term cytotoxicity assay. TRAC_CAR_ T cells were challenged daily with RAJI cells at a ratio of T cells to tumor cells of 5-to-1. Fresh tumor cells (0.2 × 10^6^ cells) were added everyday up to day 4 after pelleting and resuspended the TRAC_CAR_ T cells in fresh culture media. From day 5 onward, 5× the original number of tumor cells (10^6^ cells) was added. The antitumor activity of the TRAC_CAR_ T cells was monitored everyday by measuring the luminescence of the remaining RAJI tumor cells expressing luciferase. Cells were counted and analyzed by flow cytometry on days 0, 2, 5, and 8, and the number of CD8 (+) CAR T cells was computed and plotted as a function of time. The data are averaged +/− standard deviation from two experiments performed with cells from two different donors. Two-way ANOVA was used in panel **b** for statistical analysis (*p*-value are indicated). **c** Comparison of IL-12P70 secretion for different TRAC_CAR_ T cell groups at day 5 of the serial killing assay. **d** Comparison of CD62L MFI among CD8 (+) CAR T cells obtained for the different TRAC_CAR_ T cell groups at day 8 of the serial killing assay. The data shown in **c** and **d** were obtained from two experiments performed with cells from two different donors. On each box plot, the central mark indicates the median, the bottom and top edges of the box indicate the interquartile range and the whiskers represent the maximum and minimum data point. Source data are provided as a Source Data file

As the secretion of IL-12P70 by TILs and CAR T cells can enhance the cytolytic properties of CAR T cells in vitro and in vivo^9,20,21,49^, we asked whether the amount of IL-12P70 secretion observed during TRAC_CAR_ T cell activation in vitro (Fig. 4) was sufficient to improve antitumor activity. To test this, we compared in vitro the short-term and long-term antitumor activities of purified TRAC_CAR_ T cells (100% CAR positive T cells) with or without transgenic IL-12P70 expression. Short-term antitumor activity was characterized in vitro by using the activation protocol described in Fig. 3a (between days 10 and 12) and kinetics of tumor cell disappearance were monitored by flow cytometry. After the first tumor cell challenge, IL-12P70-expressing TRAC_CAR_ T cells elicited a faster tumor cell clearance than control TRAC_CAR_ T cells (Fig. 6a; Supplementary Fig. 7). This kinetic difference, also observed after the second tumor cell challenge, resulted in more than a 100-fold difference in residual tumor cells detected after 8 days of culture with and without IL-12P70. Thus, we hypothesized that the IL-12P70-mediated increase in tumor cell clearance rate might translate into long-term tumor cell control by TRAC_CAR_ T cells, even after repeated tumor cell challenges. We performed an in vitro serial killing assay^36^ that allowed us to repeatedly challenge TRAC_CAR_ T cells with daily doses of tumor cells for 8 consecutive days (Fig. 6b). To ensure efficient discrimination between the activity of IL-12P70-secreting TRAC_CAR_ T cells and control TRAC_CAR_ T cells, the daily doses of tumor cell were increased 5-fold (from 1× to 5×) during the last 4 days of this assay. Daily challenges of TRAC_CAR_ T cells edited by either IL2rα or PDCD1 TALEN in the absence of the IL-12P70 matrix (TRAC_CAR__CD25 and TRAC_CAR_ ΔPD1, black open circles, Fig. 6b) resulted in suboptimal tumor cell control and eventual tumor cell outgrowth. In contrast, IL-12P70-secreting TRAC_CAR_ T cells (TRAC_CAR__CD25_IL12_, red open circles, and TRAC_CAR__ ΔPD1_IL12_, blue open circles, Fig. 6b, upper panel) showed continuous and efficient tumor cell control correlated with the secretion of IL-12P70 and increased surface expression of CD62L (Fig. 6c, d). In the same in vitro assay, IL-12P70 secretion significantly improved the expansion of CD8+ TRAC_CAR_ T cells (Fig. 6b lower panel, *p* = 0024 and *p* = 0.052 for TRAC_CAR__CD25_IL12_ and TRAC_CAR__ΔPD1_IL12_, respectively). In addition, we compared the cytotoxic activity of rLV CAR_NFAT_IL12_ and AAV6-based TRAC_CAR__ΔPD1_IL12_ engineered CAR T cells (Supplementary Figs. 6e and 5f) using a similar serial killing assay. Both engineering strategies led to similar cytotoxic activity and confirmed the immunostimulatory effect of IL-12P70 on CAR T cell antitumor activity.

**Fig. 7:**
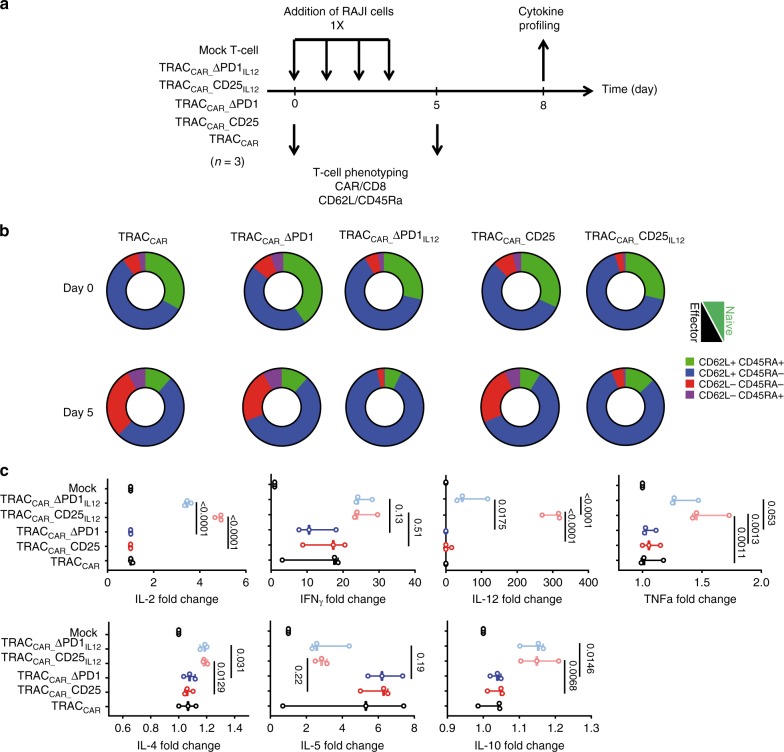
Immunoprofiling of engineered TRAC_CAR_ T cells activated by tumor cells in vitro. **a** Schematic showing the experimental design to investigate the effector and memory phenotype and cytokine secretion profile of activated engineered TRAC_CAR_ T cells. 10^6^ TRAC_CAR_ T cells were mixed with a suspension of RAJI-luc tumor cells at E:T = 5:1 in a total volume of 40 mL of Xvivo-15 media supplemented with 5% AB serum and incubated in a GREX-10 device. The mixture was incubated for 24 h before adding 2 × 10^6^ RAJI-Luc cells. The resulting cell mixture was incubated for 24 h and the same procedure was repeated to get a total of 4 RAJI cell challenges. The mixture was then incubated as is for 4 additional days and where spun down to recover the supernatant and determine the cytokine secretion profile of TRAC_CAR_ T cell. TRAC_CAR_ T cells were also analyzed by flow cytometry to determine the frequency of CD62L and CD45RA population within CD8+ CAR+ T cells (day 0 and day 5 post tumor cell-dependent activation). **b** Frequency of T cell subpopulation displaying CD62L+CD45RA+ (naive-like), CD62L+ CD45RA− (central memory), CD62L-CD45RA− (effector memory), CD62L-CD45RA+ (terminal effector) labeling and obtained at day 0 and day 5 post tumor cell-dependent activation for different engineered T cells. A linear mixed model, using lmer from the lme4 R package was used for statistical analysis. p-values are documented in Supplementary Table 1. **c** Cytokine profiling assay results obtained from supernatant of engineered T cell/RAJI cells cocultured for 5 days. The data shown in **b** and **c** represent the average of three experiments performed with cells engineered from three different donors. The mean of cytokine concentration fold change and the whiskers representing the maximum and minimum data points are illustrated in **c**. Two-way ANOVA was used in panel **c** for statistical analysis (p-value are indicated on the figures). Source data are provided as a Source Data file

In previous studies^11,50,51^, the presence of IL-12P70 at the time of T cell activation was reported to simultaneously support increased CD62L expression and enhanced effector function. To investigate this aspect in more details, we performed cytokine and surface marker profiling of TRAC_CAR_ T cells chronically challenged by RAJI cells for four days of cell culture (Fig. 7a). Comparison of CD62L and CD45RA surface markers expression indicated that IL-12P70 expressing TRAC_CAR_ T cells retained a higher frequency of naïve markers and a lower frequency of effector markers than their TRAC_CAR_ T cell counterparts (Fig. 7b and Supplementary Table 1). Consistent with earlier reports^52^, this phenomenon was correlated with a significant increase of IL-2 (*p* < 0.0001 for TRAC_CAR__CD25_IL12_ and TRAC_CAR__ΔPD1_IL12_, two-way ANOVA), TNFα (*p* = 0.0013 for TRAC_CAR__CD25_IL12_, two-way ANOVA) for and IL-10 secretion (*p* < 0.02 for TRAC_CAR__CD25_IL12_ and TRAC_CAR__ΔPD1_IL12_, two-way ANOVA) and a marked increase of IFNγ secretion by engineered cells (Fig. 7c). Interestingly, IL-12P70 secretion correlated with a significant increase of IL-4 (*p* < 0.05, for TRAC_CAR__CD25_IL12_ and TRAC_CAR__ΔPD1_IL12_, two-way ANOVA), although the fold change was negligible. In addition, IL-5 was markedly, although not significantly, decreased in IL-12P70 secreting TRAC_CAR_ T cells groups.

**Fig. 8:**
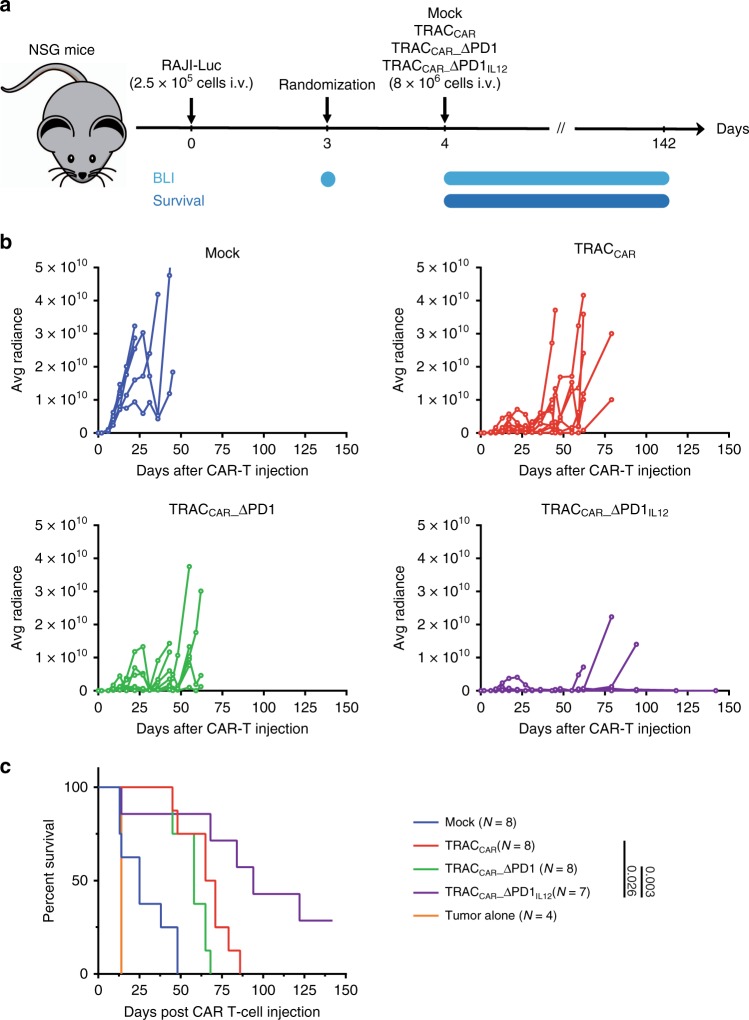
Conditional secretion of IL-12P70 increases antitumor activity of TRAC_CAR_ T cells in vivo. **a** Schematic showing the experimental design to investigate the antitumor activity of engineered TRAC_CAR_ T cells in xenograft mice model. Immunodeficient NSG mice were adoptively transferred on day 0 with RAJI-Luc-GFP tumor cells (2.5 × 10^5^ cells per animal in 100 μL of PBS i.v.). Tumor cells were allowed to expand until mice randomization, performed at day 3 on the basis of the level of tumor cell bioluminescence signal. On the same day, mice were adoptively transferred (i.v.) with 7 × 10^6^ viable mock-transduced T cells, TRAC_CAR_ T cells (*n* = 8), TRAC_CAR__ΔPD1 (*n* = 8), TRAC_CAR__ΔPD1_IL12_ (*n* = 7) T cells or tumor alone (*n* = 4). RAJI-Luc-GFP tumor cell expansion was monitored on various days by bioluminescence imaging (BLI) up to 142 days. **b** Evolution of average radiance as a function of time for the different mouse cohorts. Dotted lines correspond to the average radiance recorded for individual mouse. Comparison of tumor growth was done using the aucVardiTest function of the clinfun R package. *P*-value are indicated in the Supplementary Table 2. **c** Kaplan Meier plot obtained for different mice cohorts. Log-rank (Mantel-Cox) test was used for statistical analysis (*p*-value are indicated on the figures). Source data are provided as a Source Data file

Finally, to assess the influence of tumor cell-dependent IL-12P70 secretion in a more complex system and test the robustness of our in vitro characterization, we evaluated the antitumor activity of TRAC_CAR_ T cells in vivo using a NSG xenograft tumor model (Fig. 8). As a proof of concept, we focused on the engineering at the PDCD1 locus rather than the IL2rα locus because (1) concomitant inactivation of PD1 and secretion of IL-12 represents a dual advantage for TRAC_CAR_ T cell functions and bears high potential for clinical application and (2) the PDCD1 locus supported IL-12P70 secretion in smaller amounts than the IL2rα locus in vitro (Fig. 3h, 4b, 6c, and 7c). Thus, it was of particular interest to test whether the more moderate of the two approaches already has antitumor benefit in vivo. Consistent with earlier results, TRAC_CAR__ΔPD1_IL12_ was found to control tumor growth better (Fig. 8b, Supplementary Table 2) and to extend mice survival significantly over its TRAC_CAR_ or TRAC_CAR__ΔPD1 counterparts (Fig. 8c, *p* = 0.026 and 0.003, respectively, Log-rank Mantel cox test). This improvement of antitumor activity was not associated with weight loss in the TRAC_CAR__ΔPD1_IL12_ group within the first 45 days, except for one mouse that died without any sign of tumor relapse 14 days post tumor injection (Supplementary Fig. 8). Altogether, our results indicate that the tumor-dependent and regulated expression of IL-12P70 increases the antitumor activity of TRAC_CAR_ T cells and their capacity to accumulate (Fig. 6b), as well as decreases the frequency of effector memory cells (Fig. 6d, b)^26,53^, recapitulating the known biological and therapeutic benefits of IL-12P70^8,10,11,51,54,55^.

## Discussion

The goal of this work was to engineer smart CAR T cells that are able to sense and react to their environment in a tailored, highly regulated, and antigen-specific manner. As a proof of concept, we simultaneously repurposed TRAC along with the IL2rα or PDCD1 genes to express a CAR at the surface of primary T cells and to harness T cell engagement with tumor cell targets to promote conditional and transient secretion of IL-12P70. Using a combination of TALEN technology and AAV6 embedding promoter-less repair matrices, we efficiently delivered the CAR to the TRAC locus and to target IL-12P70 to the IL2rα and PDCD1 loci. This multiplex targeted insertion led to the disruption of PDCD1 and TRAC genes and to the non-disruptive modification of the IL2rα gene, ultimately allowing the expression of a CAR and the secretion of IL-12P70 in the media. This secretion was transient, dependent on tumor cell engagement, and followed the tight regulation patterns of CD25 and PD1 observed upon T cell activation. The levels of IL-12P70 secreted in the media were sufficient to enhance the short-term and long-term antitumor activity and the capacity of TRAC_CAR_ T cells to accumulate. In addition, our approach prevented the surface expression of PD1, one of the major checkpoints of T cell function.

Repurposing of these three genes enabled engineered TRAC_CAR_ T cells to secrete IL-12P70 in a tumor cell-dependent manner without causing commonly reported side effects in vitro^20^. Indeed, the insertion of an IL-12P70 secretion cassette under the control of the NFAT promoter in primary T cells via gammaretroviral transduction has been reported to impair T cell proliferation ex vivo and in vivo^20,48^. This lack of proliferation was linked to the gammaretrovirus mediated random insertion of IL-12P70 and to the leakiness of the NFAT-IL-12P70 secretion cassette, even in the absence of a tumor cell target (Supplementary Fig. 6 and ref. ^20^). The targeted insertion of IL-12P70 reported in this study did not impact the viability and proliferation capacity of TRAC_CAR_ T cells and did not lead to unwanted secretion of IL-12P70 in the absence of tumor cells (Fig. 4a, b, Suplementary Figs. 4d–e and 5). In the presence of tumor cells, however, TRAC_CAR_ T cells secreting IL-12P70 accumulated significantly more than their control TRAC_CAR_ T cell counterparts (Fig. 6b, *p* ≤ 0.0052 for TRAC_CAR__CD25_IL12_ and TRAC_CAR__ΔPD1_IL12_, two-way ANOVA), likely due to IL-12P70 preventing activation cell death^56^. This finding indicates that the IL2rα and PDCD1 regulatory elements and patterns of activation can efficiently prevent unwanted leakiness of IL-12P70 secretion, which can be deleterious for CAR T cell expansion^20,57^.

Although this targeted insertion strategy avoids the common drawbacks of gammaretrovirus-dependent or lentivirus-dependent random transgene insertions^58^, it may have some downsides. Indeed, although correct targeted matrix insertion occurred in the majority of modified cells without detectable random matrix insertion, a minority of them harbored TRAC_CARm_ insertion at IL2Rα or PDCD1 loci and IL2Rα_IL12P70m_ or PDCD1_IL12P70m_ insertion at the TRAC locus. These homology-independent integrations were also recently reported after simultaneous editing of TRBC and TRAC loci^34^ and a series of different pairs of genes using CRISPR-CAS9 and non-virally delivered template DNA^31^. As demonstrated^31^, these marginal events are unlikely to promote transgene expression because of the lack of exogenous promoter in the matrices and given the potential mismatches between targeted loci and transgenes’ open reading frames. Thus, although this phenomenon warrants a systematical investigation prior to clinical use, we believe that this phenomenon is unlikely to promote aberrant adverse events. To reduce the risks posed by homology-indepent integrations, an alternative may be to separate the engineering of the two loci in time. For example, performing a sequential TALEN treatment would prevent simultaneous double strand-break formation at 2 loci and limit the risk of off-target insertion. This approach could also be beneficial to prevent translocations. To that matter, although accurate quantification of translocation was limited by the low sensitivity of FISH analysis, our results suggest that there is no detectable proliferative advantage conferred by PD1/TRAC translocations during cell expansion. Further work should assess this point before clinical applications.

Our work shows that engineered TRAC_CAR_ T cells are able to sense subtle differences in tumor cell concentration and to secrete IL-12P70 accordingly. The level of IL-12P70 secretion correlated with the quantity of tumor cells present in the media in a saturated fashion (Fig. 4b). In addition, secretion appeared to increase rapidly upon tumor cell engagement and decreased at a rate similar to that observed for tumor disappearance (Figs. 4a and 6a). Thus, engineered TRAC_CAR_ T cells could be considered as a rheostat that quantitatively and rapidly adapt their IL-12P70 secretion output as a function of tumor cell concentration input. This fine reactivity is likely due to the simultaneous repurposing of TRAC and either IL2rα or PDCD1, which enables successful repuposing of their regulatory elements and recapitulates the natural and exquisite regulation of the TCR pathway. Therefore, our work shows that it is possible to modify interconnected genetic elements to rewire the cellular circuitry and transform biological inputs into relevant therapeutic outputs.

The low and transient levels of IL-12P70 secreted by engineered TRAC_CAR_ T cell were sufficient to elicit greater antitumor activity than control TRAC_CAR_ T cells in vitro and in vivo. The activation-induced secretion of IL-12p70 also correlated with a increased level of IL-2, TNFα, IL-10 and cell surface presentation of CD62L, a marker expressed in the central memory, stem memory, and naive T cells. This phenotype was previously reported for T cells activated in the presence of IL-12P70^11,50–52^. These positive effects are likely to be enhanced in vivo by the ability of IL-12P70 to recruit innate immune cells, which synergistically improve CAR T cell antitumor activity^10,59^. This particular aspect should be further investigated in an immunocompetent mouse model.

Engineering CAR T cells with customized therapeutic response programs was first reported using single therapeutic transgenes under the control of generic exogenous promoters. However, the constitutive nature of transgene expression under these conditions and the potential toxicity associated with these engineering strategies led to the development of inducible expression systems that take advantage of drug-inducible or tumor-inducible promoters and, more recently, to the engineering of synthetic Notch receptors^48,60–63^. Here we chose a different approach that takes advantage of the highly regulated nature of immune pathways to endow CAR T cells with customized activity. The precise repurposing of endogenous genes reported in this work allows users to bypass the need for an exogenous promoter and exploits optimized regulatory networks to express a therapeutic transgene of interest in a conditional fashion. More importantly, thanks to the availability of high content databases reporting temporal gene transcription and protein expression in various physiological environments^64,65^, one can customize the amplitude and kinetics of therapeutic transgene delivery by choosing the gene to rewire in a strategic manner.

Endogenous gene network repurposing becomes now possible with the recent advances of gene editing technics^66^ and DNA template vectorization methods (single and double DNA templates^31,34,67–69^, and AAV6-embeded template^26,29,32,70^). Our work illustrates the therapeutic potential of this approach by rewiring different elements of the TCR activation pathway, but is not limited to it. Indeed, it can be extended to other gene networks to sense a wide variety of relevant inputs (e.g., tumor microenvironment biomarkers such as acidosis, oxygen, and arginine deprivation) and to translate them into different genetically encoded therapeutic outputs. Thus, our work paves the way for the repurposing of genetic pathways to generate smart CAR T cells that are able to integrate complex environmental inputs and to generate custom outputs in a highly regulated and specific manner.

## Methods

### Biological materials

Cryopreserved human PBMCs were acquired from ALLCELLS (cat # PB006F) and used in accordance with Cellectis IRB/IEC-approved protocols. PBMCs were cultured in X-vivo-15 media (obtained from Lonza Group, cat # BE04-418Q), containing IL-2 (obtained from Miltenyi Biotech, cat # 130-097-748), and human serum AB (obtained from Seralab, cat # GEM-100-318). Human T activator CD3/CD28 from Life Technologies/Thermo Fisher Scientific (cat # 11132D) was used to activate T cells. TRAC_CAR_ T cells were purified using a CD34 MicroBead kit and a MACS® LD-column from Miltenyi Biotech (cat # 130-046-702 and cat # 130-042-901, respectively). Post-staining was carried out with antibody QBEND10-APC from R&D Systems (cat # FAB7227A). All other antibodies used and the corresponding concentrations are summarized in Table 1.

**Table 1:** Antibodies used in the study

Antibody	Dilution	Fluorophore	Manufacturer	Catalogue #
Cd3	1:20	Vioblue	Miltenyi	130-094-363
Qbend10	1:10	APC	R&D systems	FAB7227A
Qbend10	1:10	FITC	R&D systems	FAB7227G
ΔLNGFR	1:20	PE	Miltenyi	130-113-421
ΔLNGFR	1:20	Vioblue	Miltenyi	130-112-606
Pd1	1:20	PE	Miltenyi	130-117-384
Cd25	1:20	PE	Miltenyi	130-091-024
Cd20	1:20	APC	Miltenyi	130-108-290
Cd19	1:20	FITC	Miltenyi	130-113-168
Cd22	1:20	PE	Miltenyi	130-105-056
Cd8	1:20	FITC	Miltenyi	130-098-059
CD62L	1:20	APC	Miltenyi	130-102-931
CD45RA	1:20	Vioblue	Miltenyi	130-117-742

### Targeted integration of CAR and IL-12P70 constructs

The double targeted integration at the TRAC and PDCD1 or IL2rα loci were performed as follows: PBMC cells were thawed, washed, resuspended, and cultivated in X-vivo-15 complete media (X-vivo-15, 5% AB serum, 20 ng/mL IL-2). One day later, the cells were activated with Dynabeads human T activator CD3/CD28 (25 µL of beads/10^6^ CD3 positive cells) and cultivated at a density of 10^6^ cells/mL for 3 days in X-vivo complete media at 37 °C in the presence of 5% CO_2_. The cells were then split into fresh complete media and transduced/transfected the next day according to the following procedure. On the day of transduction-transfection, the cells were first de-beaded by magnetic separation (EasySep), washed twice in Cytoporation buffer T (BTX Harvard Apparatus, Holliston, Massachusetts), and resuspended at a final concentration of 28E^6^ cells/mL in the same solution. This cellular suspension was mixed with 5 µg mRNA encoding TRAC TALEN arms in the presence or absence of 15 µg of mRNA encoding arms of either PDCD1 or IL2rα TALEN (please refer to Supplementary Data 2 for the TALEN arm mRNA sequences and Table 2 for the target DNA sequences) in a final volume of 200 µl. Transfection was performed using Pulse Agile technology by applying two 0.1 mS pulses at 3000 V/cm followed by four 0.2 mS pulses at 325 V/cm in 0.4 cm gap cuvettes and in a final volume of 200 µL of Cytoporation buffer T (BTX Harvard Apparatus, Holliston, Massachusetts). The electroporated cells were then immediately transferred to a 12-well plate containing 1 mL of prewarmed X-vivo-15 serum-free media and incubated at 37 °C for 15 min The cells were then concentrated to 8E^6^ cells/mL in 250 µL of the same media in the presence of AAV6 particles (MOI = 3E^5^ vg/cells) comprising the donor matrices (Supplementary Data 1) in 48-well regular treated plates. After 2 h of culture at 30 °C, 250 µL of Xvivo-15 media supplemented by 10% AB serum and 40 ng/mL IL-2 was added to the cell suspension, and the mix was incubated for 24 h under the same culture conditions. One day later, the cells were seeded at a density of 10^6^ cells/mL in complete X-vivo-15 media and cultivated at 37 °C in the presence of 5% CO_2_.

**Table 2:** TALEN targets used in the study

TALEN	Sequence targeted
TRAC	**T**TGTCCCACAGATATCCagaaccctgaccctgCCGTGTACCAGCTGAG**A**
IL2rα	**T**ACAGGAGGAAGAGTAGaagaacaatctAGAAAACCAAAAGAAC**A**
PDCD1	**T**ACCTCTGTGGGGCCATctccctggcccccaaGGCGCAGATCAAAGAG**A**

For all sequences, the left and right binding sites are indicated by uppercase letters, and spacers are indicated with lower-case letters. The T0 and A0 nucleotides are indicated in bold

### Lentivirus-based engineering of T cells

PBMC cells were thawed, washed, resuspended, and cultivated in X-vivo-15 complete media (X-vivo-15, 5% AB serum, 20 ng/mL IL-2). One day later, the cells were activated with Dynabeads human T activator CD3/CD28 (25 µL of beads/10^6^ CD3 positive cells) and cultivated at a density of 10^6^ cells/mL for 3 days in X-vivo complete media at 37 °C in the presence of 5% CO_2_. The cells were incubated on retronectin coated plates, with 2 independent lentiviral particles encoding CD22 CubiCAR (MOI 5) and IL-12P70 under the control of 6 NFAT promoter followed by a minimal IL-2 promoter (MOI 0.25^20,48^) in Xvivo-15 media and were supplemented by 1 volume of Xvivo 10% Ab serum, 40 ng/mL IL-2 and incubated overnight. One day later, cells were seeded at a density of 10^6^ cells/mL in complete X-vivo-15 media and cultivated at 37 °C in the presence of 5% CO_2_.

### Isolation of TRAC_CAR_ T cells using magnetic separation

TRAC_CAR_ T cells were purified 4 days after transduction using a CD34 MicroBead kit and MACS-LD column from Miltenyi Biotech as previously described^36^. Briefly, 5–10 × 10^7^ T cells were incubated with 100 µL of FC-blocking reagent and 125 µL QBEND10 coated magnetic beads for 30 min in a final volume of 500 µL CliniMACS Buffer. The cellular suspension was then loaded onto a MACS® LD-Column attached to a conventional Myltenyi magnet and washed three times with 1 mL of CliniMACS Buffer. The column was eventually removed from the magnet, and the TRAC_CAR_ T cells were eluted in 3 mL of the same buffer. One round of purification was sufficient to obtain a homogeneous population of CubiCAR T cells (purity > 96%).

### Cytogenic characterization of TRAC_CAR_ T cells

Engineered TRAC_CAR_ T cells expanded for a total of 17 days in G-Rex were subjected to TLA, FISH, and karytope analysis.

### Karyotype and fluorscence in situ hybridization (FISH)

TRAC_CAR_ T cells were incubated with RAJI cells (E/T ratio = 1) for 3 days to allow them to divide. Dividing TRAC_CAR_ T cell were then fixed and hybridized with TRAC, PDCD1, or IL2Rα specific fluorescent probes according to FISH standard procedures (Supplementary Materials section). The same procedure was performed with MRC-5, a human fibroblast cell line considered as a reference diploid cell line by the European Medicine Agency and commonly used as a negative control for gene fusion detection by FISH. 100 metaphasic nuclei from the MRC-5 negative control and 500 metaphasic nuclei from TRAC_CAR_ T cells were screened for fusions of the TRAC gene (14q11.2) with either the PDCD1 gene (2q37.3) or IL2Rα gene (10p15.1).

Regarding Karyotype, a total of one hundred metaphases were studied for each sample. Eighty (80) metaphases were screened for aberrations of the TRAC and PDCD1 chromosomal loci (14q11.2 and 2q37) or TRAC and IL2Rα chromosomal loci (14q11.2 and 10p15.1), the ploidy level (hyperploidy, hypoploidy, polyploidy) and the modal chromosome number determination but also, for any gross structural abnormalities including chromosome breaks and gaps among others (dicentrics, acentric fragments). Twenty additional metaphases were karyotyped according to the International System for Chromosome Nomenclature (ISCN) based on their G-banding pattern for the detection of any specific structural chromosomal aberration including deletions, translocations involving 14q11.2/2q37 or 14q11.2/10p15.1, inversions, duplications and additions. The modal chromosome number of the 2 tested samples, originating from the same T cell donor, was defined as 46 chromosomes (22 pairs of autosomes and one pair of sex chromosomes) and the sex of the sample was female. Experiments were performed by Cleancells (Bouffere, Frnace).

### Targeted locus amplification (TLA)

TLA analysis of engineered TRAC_CAR_ T cell was performed by Cergentis (Utrecht, Netherlands) according to ref. ^40^ using a set of multiple primers described in Supplementary Tables 3–4. Regarding TRAC_CAR__ΔPD1_IL12_ analysis, 2 primer sets were designed on the genome in the TRAC and PDCD1 loci (Supplementary Table 3, sets 1–4), and 2 primer sets were designed on TRAC_CARm_ and PDCD1_IL12m_, Supplementary Table 3, sets 5–8). Regarding TRAC_CAR__CD25_IL12_ analysis, 2 primer sets were designed on the genome in the TRAC and IL2Rα loci (Supplementary Table 4, sets 1–4), and 2 primer sets were designed on TRAC_CARm_ and IL2Rα_IL12m_, 2 sets per matrix (Supplementary Table 4, sets 5–8). The primer sets were used in individual TLA amplifications. PCR products were purified and library prepped using the Illumina Nextera flex protocol and sequenced on an Illumina sequencer. Reads were mapped using BWA-SW, version 0.7.15-r1140, settings bwasw -b 7. The NGS reads were aligned to the matrices sequence and host genome. The human hg19 genome was used as host reference genome sequence. Integration sites were detected based on a coverage peaks in the genome and on the identification of fusion-reads between the matrices sequence and the host genome.

### Oligo capture assay

To explore the specificity of TALEN targeting, an Oligo capture assay (OCA) derived from ref. ^43^ was used. The Oligo capture assay detected the on-target and off-target site of TALEN through the incorporation of small duplex oligodeoxynucleotides (dsODNs) into the double-stranded breaks (DSBs) induced by the TALEN nuclease activity. These labeled DSBs were then mapped at nucleotide resolution by amplification of sequences bearing an integrated dsODN and high throughput DNA sequencing. In brief, cells were transfected with TRAC and PDCD1 TALEN and dsODN and propagated for about 6 days. Genomic DNA was harvested from these cells, randomly sheared with a sonicator and end-repaired/A-tailed. Next-generation sequencing Y-adapters (TruSeq Annealed Adapter) that contain the P5 sequence were ligated to the ends of this DNA. Nested, anchored PCR using dsODN-specific and adapter-specific primers were used to specifically amplify the plus and minus strand of DNA fragments that incorporated the dsODN. The first round of PCR used the following adapter-specific (P5_1) and dsODN-specific primers. The second round of PCR used the following adapter-specific (P5_2) and dsODN-specific primers along with a third oligo P7 that added the barcode and P7 sequence to the ends of the DNA. Equal amounts of these separate PCRs were pooled together and sequenced using Illumina MiSeq (2 × 150 bp). The resulting sequences were then mapped on human genome to identify potential offsite candidate. Potential off-site candidate identified by OCA were then verified using genomic DNA obtained from T cells transfected by PDCD1 and TRAC in the absence of dsODN. Genomic DNA was amplified using off-site candidate-specific PCR primer pairs and the resulting amplicons were analyzed by high throughput DNA sequencing to determine the frequency of Indels genetrated the TALEN treatment.

### Activation-dependent expression of ΔLNGFR and IL-12P70

Engineered TRAC_CAR_ T cells were recovered from the transfection-transduction process described above and seeded at 10^6^ cells/mL alone or in the presence of RAJI tumor cells (Effector: target, E:T = 1:1) in a final volume of 100 µL of X-vivo-15 media supplemented with 5% AB serum. Cells were collected every day for 8 days after activation, labeled, and analyzed by flow cytometry using specific antibodies. From the same experimental set up, 100 µL of supernatant was collected each day to measure IL-12P70 secretion in the media using an IL-12P70 specific ELISA kit by R&D systems according to the manufacturer’s protocol.

### Serial killing assay

To assess the antitumor activity of engineered TRAC_CAR_ T cells, a serial killing assay was performed using a protocol similar to that reported in^36^. TRAC_CAR_ T cells were mixed with a suspension of 2 × 10^5^ RAJI-luc tumor cells at E:T = 5:1 in a total volume of 1 mL of Xvivo-15 media supplemented with 5% AB serum. The mixture was incubated for 24 h before determining the luminescence of 25 µL of cell suspension using 25 µL of ONE-Glo reagent (Promega). The cell mixture was then spun down, and the old media was discarded and substituted with 1 mL of fresh complete Xvivo-15 media containing 2 × 10^5^ RAJI-Luc cells. The resulting cell mixture was incubated for 24 h. This protocol was repeated up to 4 days. The same protocol was used from days 5 to 8 with the daily addition of 5 times more RAJI-Luc cells. A similar protocol was used to investigate the effector and memory phenotype and cytokine secretion profile of activated engineered TRAC_CAR_ T cells. 10^6^ TRAC_CAR_ T cells were mixed with a suspension of RAJI-luc tumor cells at E:T = 5:1 in a total volume of 40 mL of Xvivo-15 media supplemented with 5% AB serum and incubated in a GREX-10 device. The mixture was incubated for 24 h before adding 2 × 10^6^ RAJI-Luc cells. The resulting cell mixture was incubated for 24 h and the same procedure was repeated to get a total of 4 RAJI cell challenges. The mixture was then incubated as is for 4 additional days and where spun down to recover the supernatant and determine the cytokine secretion profile of TRAC_CAR_ T cell. TRAC_CAR_ T cells were also analyzed by flow cytometry to determine the frequency of CD62L and CD45RA population with CD8+ CAR + T cells (at day 0 and 5 post activation).

### In vivo experiment using NGS xenograft model

All procedures involving animals were performed in accordance with regulations and established guidelines and were reviewed and approved by the Cellectis Institutional Animal Care and Use Committee (IACUC), as well as by the Animal Ethical Committee at Mispro-Biotech (New York, NY).

### Tumor-dependent secretion of IL-12P70 in vivo

A total of 40 immunodeficient NSG mice (NOD.Cg-*Prkdc*^*scid*^ *Il2rg*^*tm1Wjl*^/SzJ, the Jackson Laboratory), were received and acclimatized for a week. NSG mice were then injected with 0.5 × 10^6^ CD22+ RAJI and CD22− RAJI tumor cells on left and right flank, respectively. The tumor cells were allowed to expand until mouse randomization, which was performed at day 4 based on the degree of tumor cell outgrowth, measured using bioluminescence imaging (BLI) (XenoLight D-luciferin (PerkinElmer). The next day, mice were adoptively transferred (i.v.) with either 8 × 10^6^ viable mock-transduced T cell or 8 × 10^6^ viable TRAC_CAR_ positive T cells (7 mice per group, total 5 groups). The mice were then re-imaged at day 6 of TRAC_CAR_ T cell injection and sacrificed to harvest serum, spleen, and right and left tumors. The splenocytes were prepared by mechanical disruption of the organ followed by 70 µm-filtering and a Ficoll density gradient purification. The cell suspensions were analyzed by flow cytometry. The tumors were dissociated using Miltenyi’s human tumor dissociation buffer according to the manufacturer’s protocol. The cells were then filtered using a 40 µm filter and analyzed by flow cytometry.

### Antitumor activity of TRAC_CAR_ T cell in vivo

A total of 35 immunodeficient NSG (NOD.Cg-*Prkdc*^*scid*^ *Il2rg*^*tm1Wjl*^/SzJ, the Jackson Laboratory) mice were adoptively transferred on day 0 with RAJI-Luc-GFP tumor cells (2.5 × 10^5^ cells per animal in 100 μL of PBS i.v.). Tumor cells were allowed to expand until mice randomization, performed at day 3 on the basis of the level of tumor cell bioluminescence signal . On the same day, mice were adoptively transferred (i.v.) with 7 × 10^6^ viable Mock-transduced T cells, TRAC_CAR_ T cells, TRAC_CAR__ΔPD1, TRAC_CAR__ΔPD1_IL12_ T cells or no T cells. This amount of cells correspond to the actual CAR positive cell numbers injected. The different group were constituted by 8 mice for except TRAC_CAR__ΔPD1_IL12_ T cells (*N* = 7) and tumor alone group (*N* = 4). RAJI-Luc-GFP tumor cell expansion was monitored on various days by bioluminescence imaging (BLI) using XenoLight D-luciferin (PerkinElmer), injected i.p. in animals (150 μL, 10 mg/mL stock solution) before induction of narcosis (isoflurane). Data acquisition and was performed 5 min Xenolight D-luciferine injection using a Spectrum-CT apparatus (Perkin Elmer) interfaced to Living Image software (Caliper). Average Radiance was determined and plotted as a function of time to reflect tumor growth curve.

## Supplementary information

Supplementary Information

Supplementary Data 1

Supplementary Data 2

## Data Availability

The authors declare that all relevant data are available in the Source Data file provided in the Supplementary Information files

## Source data

Source Data
