# Author Correction: Repurposing endogenous immune pathways to tailor and control chimeric antigen receptor T cell functionality

**DOI:** 10.1038/s41467-020-16301-w

**Published:** 2020-05-07

**Authors:** Mohit Sachdeva, Brian W. Busser, Sonal Temburni, Billal Jahangiri, Anne-Sophie Gautron, Alan Maréchal, Alexandre Juillerat, Alan Williams, Stéphane Depil, Philippe Duchateau, Laurent Poirot, Julien Valton

**Affiliations:** 1 Cellectis, Inc., 430 East 29th Street, New York, NY 10016 USA; 2 Cellectis, 8 rue de la Croix Jarry, 75013 Paris, France

**Keywords:** Cytokines, Immunotherapy, Translational immunology, Cancer

Correction to: *Nature Communications* 10.1038/s41467-019-13088-3, published online 13 November 2019.

The original version of this Article contained an error in the fifth sentence of the first paragraph of the “Targeted integration of CAR and IL-12P70 constructs” section of the “Methods” section, which incorrectly read “This cellular suspension was mixed with 5 µg mRNA encoding TRAC TALEN arms (please refer to Table. 1 for relevant sequences) in the presence or absence of 15 µg of mRNA encoding arms of either PDCD1 or IL2rα TALEN (please refer Table 2 for relevant sequences) in a final volume of 200 µl”. The correct version states “This cellular suspension was mixed with 5 µg mRNA encoding TRAC TALEN arms in the presence or absence of 15 µg of mRNA encoding arms of either PDCD1 or IL2rα TALEN (please refer to Supplementary Data 2 for the TALEN arm mRNA sequences and Table 2 for the target DNA sequences) in a final volume of 200 µl”. This error, together with the inadvertently missing mRNA information in the form of Supplementary Data 2, has been corrected in both the PDF and HTML versions of the Article.

In addition, sequence information on AAV6 repair matrices and TALEN amino acid sequences has been inadvertently omitted from the original version of the Article. This is now provided as Supplementary Data 1 associated with the Article, which is now called out in the “Methods” section “Targeted integration of CAR and IL-12P70 constructs” as follows: “The cells were then concentrated to 8E6 cells/mL in 250 µL of the same media in the presence of AAV6 particles (MOI = 3E5 vg/cells) comprising the donor matrices (Supplementary Data 1) in 48-well regular treated plates”. This phrase replaces the earlier version, which does not mention this data and reads as “The cells were then concentrated to 8E6 cells/mL in 250 µL of the same media in the presence of AAV6 particles (MOI = 3E5 vg/cells) comprising the donor matrices in 48-well regular treated plates”. This has now been corrected in both the PDF and HTML versions of the Article.

