# Supplementary material for: Repurposing endogenous immune pathways to tailor and control chimeric antigen receptor T cell functionality
Source: Nat Commun. 2019 Nov 13;10:5100. doi: 10.1038/s41467-019-13088-3 (PMC6853973; doi:10.1038/s41467-019-13088-3)
Supplement: Supplementary file 1 — Supplementary Information [file 41467_2019_13088_MOESM1_ESM.pdf]

**Repurposing Endogenous Immune Pathways to Tailor and Control Chimeric Antigen Receptor T-cell Functionality**

Sachdeva, M *et al*.

Supplementary Figures

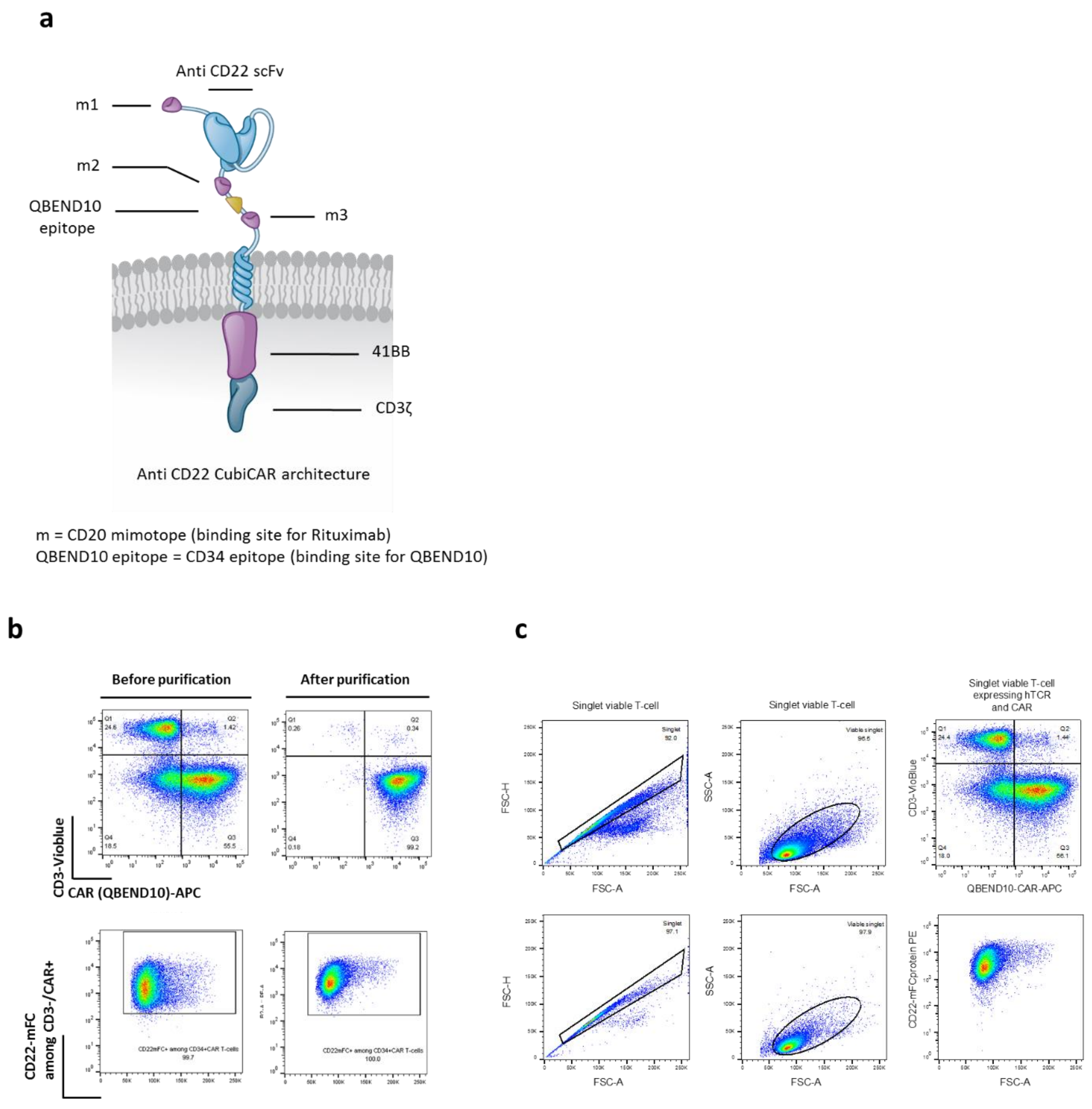


**Supplementary Figure 1. $TRAC_{CAR}$ T-cells are readily enriched by QBEND10-coated magnetic beads. a** Schematic representation of the CubiCAR architecture[1]. The cubiCAR is a tri-fonctional CAR architecture enabeling the depletion, enrichment and detection of $TRAC_{CAR}$ T-cell. It is composed of 3 CD20 mimotopes (enabling the Rituximab-dependent depletion of $TRAC_{CAR}$ T-cells), 1 QBEND10 epitope (enabling the purification and detection of $TRAC_{CAR}$ T-cells), an ScfV specific for CD22, a CD8 hinge and transmembrane domain, a 4-1BB and a CD3ζ proliferation and activation domains, respectively. **b** Flow cytometry analysis

of $TRAC_{CAR}$ T-cells obtained before and after purification using QBEND10 coated beads purification. The top and bottom panels show detection of $TRAC_{CAR}$ T-cells using a CD3-Vioblue/QBEND10-APC antibody mix and a mouseFC-CD22/anti-mouseFC antibody mix, respectively. **c** Gating strategy used to detect CD3 (-) and CAR (+) expression among viable singlet T-cells.

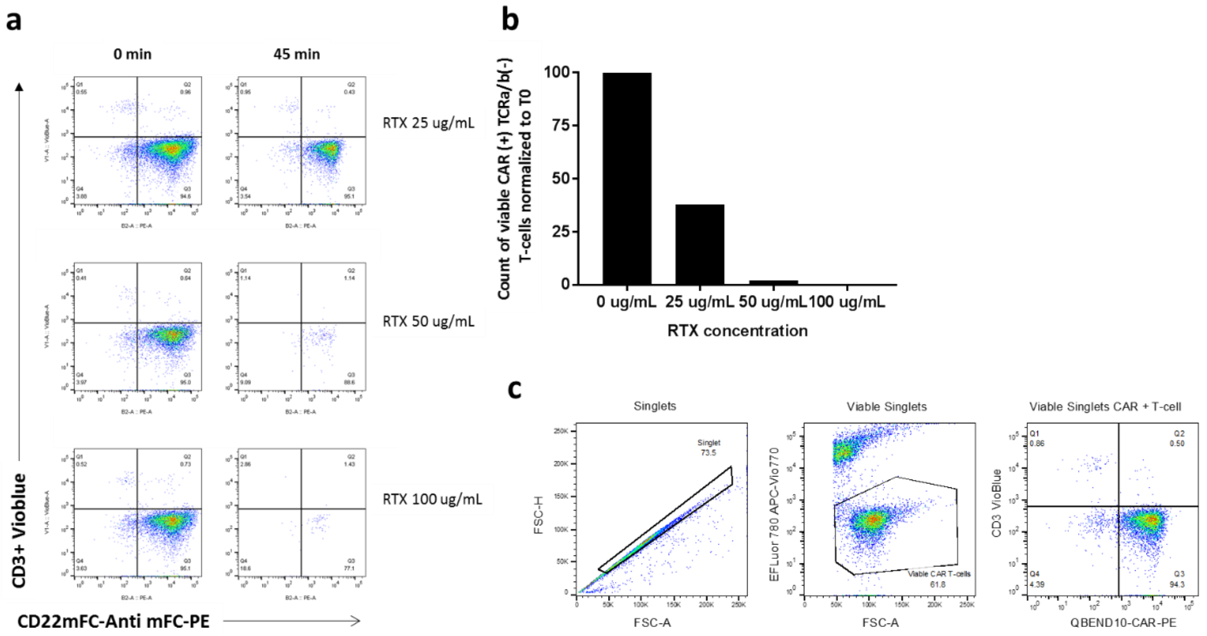


**Supplementary Figure 2. Purified $TRAC_{CAR}$ T-cells are efficiently depleted by RTX**. Purified $TRAC_{CAR}$ T-cells were incubated for 45min at 37°C in the presence of 0, 25, 50, or 100 µg/mL RTX and complement. Cells were recovered and labeled with CD3 and an anti-mFC CD22 recombinant protein (which was subsequently labeled with a secondary anti-mouse FCY-PE antibody). **a** representative flow cytometry results. **b** Frequency of remaining CAR(+) and CD3(-) T-cells relative to the no-RTX control, plotted as a function of RTX concentration. **c** Gating strategy used determine the frequency CAR(+) and CD3(-) T-cells among viable singlets obtained with or without Rituximab treatment. Source data are provided as a Source Data file.

## 3a

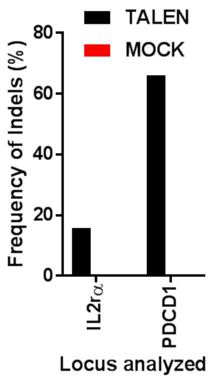


## 3b

| Offsite # | Sequence | Chromosome # | Start | End | Gene | Position | Distance from Gene (bp) | Gene description | Frequency of Indels detected: Mock-transfected T-cells | Frequency of Indels detected: TALEN Transfected T-cells |
|---|---|---|---|---|---|---|---|---|---|---|
| OS1 | TCTCTGTGCTCTGTGCAtggagtggggcccACTCATCTGTGGTTTAA | chr2 | 87192974 | 87193007 | MIR4771-1 | - | 1796 | MicroRNA 4771-1 | 0.04 | 0.04 |
| OS2 | TTAAACCACAGATGAGTgggccccactccaTGCACAGAGCACAGAGA | chr2 | 111772914 | 111772952 | ANAPC1 | Gene (Intron) | 0 | Anaphase promoting complex subunit 1 | 0.04 | 0.04 |
| OS3 | TTGCTCCCCAGAGATATtcggcaatgtccagAGACATTTGTGAGACAA | chr16 | 17344051 | 17344096 | XYLT1 | Gene (Intron) | 0 | Xylosyltransferase 1 | 0.01 | 0.01 |
| OS4 | ATATGGCTGGTTCCCCAacatgcctcaccctcATCTATATCCTTTGGCAGCTCACAGGTG | chr1 | 160739350 | 160739363 | SLAMF7 | Gene (exon) | 0 | SLAM family member 7 | 0.01 | 0.01 |
| OS5 | TTCTCCCACAGAACTGGaccacatacttCTGGGTATTAGTTGAGA | chr10 | 123100507 | 123100551 | - | - | - | - | 0 | 0 |
| OS6 | CACGGTGGTGGGGCCGCtgggcaggagagaggGGCTGCCTGACCCTGCCGTGT | chr10 | 133235801 | 133235808 | UTF1 | - | 4246 | Undifferentiated embryonic cell transcription factor 1 | 9.12 | 7.42 |

Potential TRAC/PDCD1 offsite candidates identified by Oligo Capture Assay

Assessment of offsite candidates editing by High Throughput DNA Sequencing

**Supplementary Figure 3. Characteristics of IL2rα, PDCD1 and TRAC/PDCD1 TALEN treatments**. **a** Frequency of insertions and deletions (Indels) found at IL2rα and PDCD1 loci 4 days after TALEN treatment. Following TALEN treatment, genomic DNA from the transfected cells was recovered, amplified using IL2rα and PDCD1-specific primer pairs and the resulting PCR amplicons were analyzed by high throughput DNA sequencing. **b** Characteristics of TRAC/PDCD1 TALEN candidate off-sites identified by Oligo Capture Assay and determination of indels frequency promoted by TRAC/PDCD1 TALEN or mock treatment of T-cells. Data presented were obtained from on transfection experiment. Source data are provided as a Source Data file.

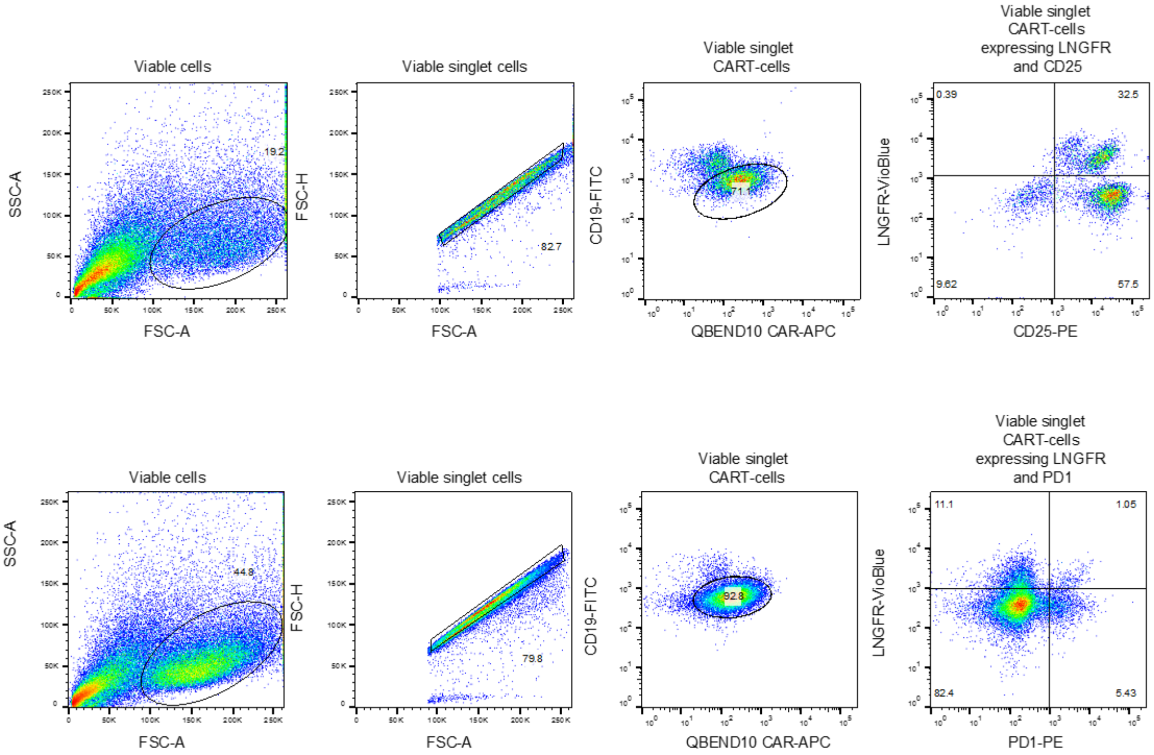


**Supplementary Figure 4. Efficient translation of tumor cell engagement by TRAC$_{CAR}$ T-cell into IL-12P70 secretion *in vitro*. Top panel** schematic showing the gating strategy to assess the extent of ΔLNGFR and CD25 surface expression among CAR (+) viable singlet T-cells, 48 hours after tumor cell-dependent activation. **Bottom panel** schematic showing the gating strategy to assess the extent of ΔLNGFR and PD1 surface expression among CAR (+) viable singlet T-cells, 48 hours after tumor cell-dependent activation.

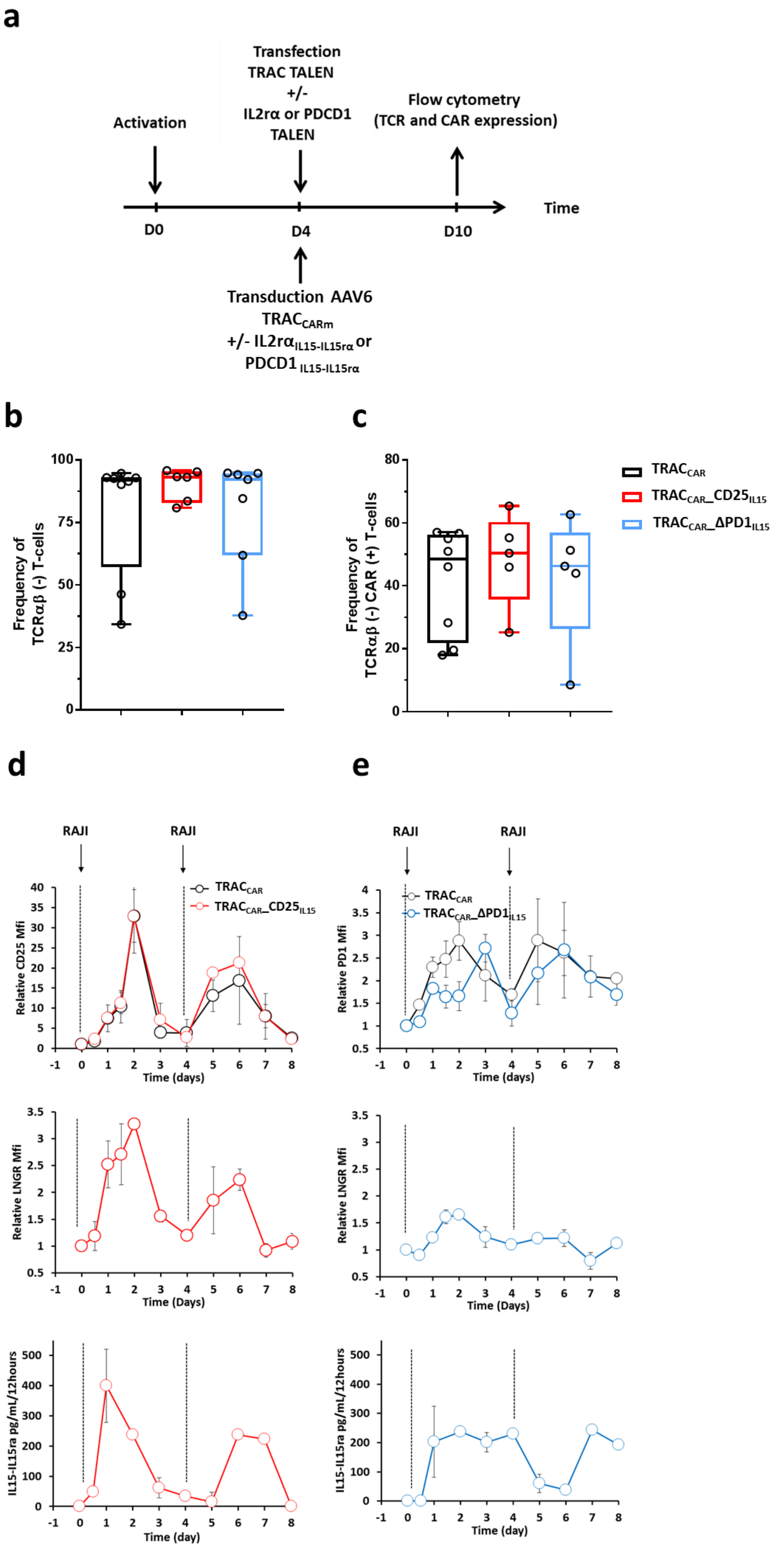
a
Activation
Transfection
TRAC TALEN
+/-
IL2rα or PDCD1
TALEN
Flow cytometry
(TCR and CAR expression)
D0
D4
D10
Time
Transduction AAV6
TRAC_CARm
+/- IL2rα_IL15-IL15rα or
PDCD1_IL15-IL15rα
b
Frequency of
TCRαβ (-) T-cells
c
Frequency of
TCRαβ (-) CAR (+) T-cells
TRAC_CAR
TRAC_CAR_CD25_IL15
TRAC_CAR_ΔPD1_IL15
d
e
RAJI
RAJI
Relative CD25 Mfi
Time (days)
Relative PD1 Mfi
Relative LNGR Mfi
Time (Days)
IL15-IL15ra pg/mL/12hours
Time (day)

**Supplementary Figure 5. Repurposing of T-cell immune pathways to secrete IL-15-IL15rα. a** Schematic showing the strategies used to repurpose TRAC along with the IL2rα or PDCD1 loci to generate $TRAC_{CAR}$, $TRAC_{CAR}$_$CD25_{IL15}$, and $TRAC_{CAR}$_$\Delta PD1_{IL15}$ T-cells from wild-type T-cells. The experimental design for multiplex repurposing of TRAC, IL2rα or PDCD1 and analysis of the resulting engineered $TRAC_{CAR}$ T-cells is illustrated. **b** and **c** Frequency of TCRαβ (-) T-cells and of TCRαβ (-) CAR (+) T-cell observed by flow cytometry, respectively, 60 hours post TALEN mRNA electroporation and AAV6 transduction. Each point represents one experiment performed with a given donor. n=8 for $TRAC_{CAR}$, n=6 for $TRAC_{CAR}$_$CD25_{IL15}$, and n=7 for $TRAC_{CAR}$_$\Delta PD1_{IL15}$ in panel **b** and n=8 for $TRAC_{CAR}$, n=5 for $TRAC_{CAR}$_$CD25_{IL15}$, and n=5 for $TRAC_{CAR}$_$\Delta PD1_{IL15}$ in panel **c**. On each box plot illustrated in **b** and **c**, the central mark indicates the median, the bottom and top edges of the box indicate the interquartile range and the whiskers represent the maximum and minimum data point. **d-e** Kinetics of CD25 and PD1 expression (top), ΔLNGFR expression (middle), and IL-15-IL15rα secretion (bottom panel) in purified $TRAC_{CAR}$ T-cells after two consecutive tumor cell challenges. Engineered $TRAC_{CAR}$ T-cells were activated by RAJI cells at Day 0 and Day 4 and analyzed by flow cytometry for 8 days. IL-15-IL15rα secreted in cell culture supernatants was quantified by ELISA and plotted as a function of time. The arrows indicate the times of tumor cell addition. The data shown represent the average of two experiments performed with cells from two different donors. The mean +/- standard deviation is represented. Source data are provided as a Source Data file.

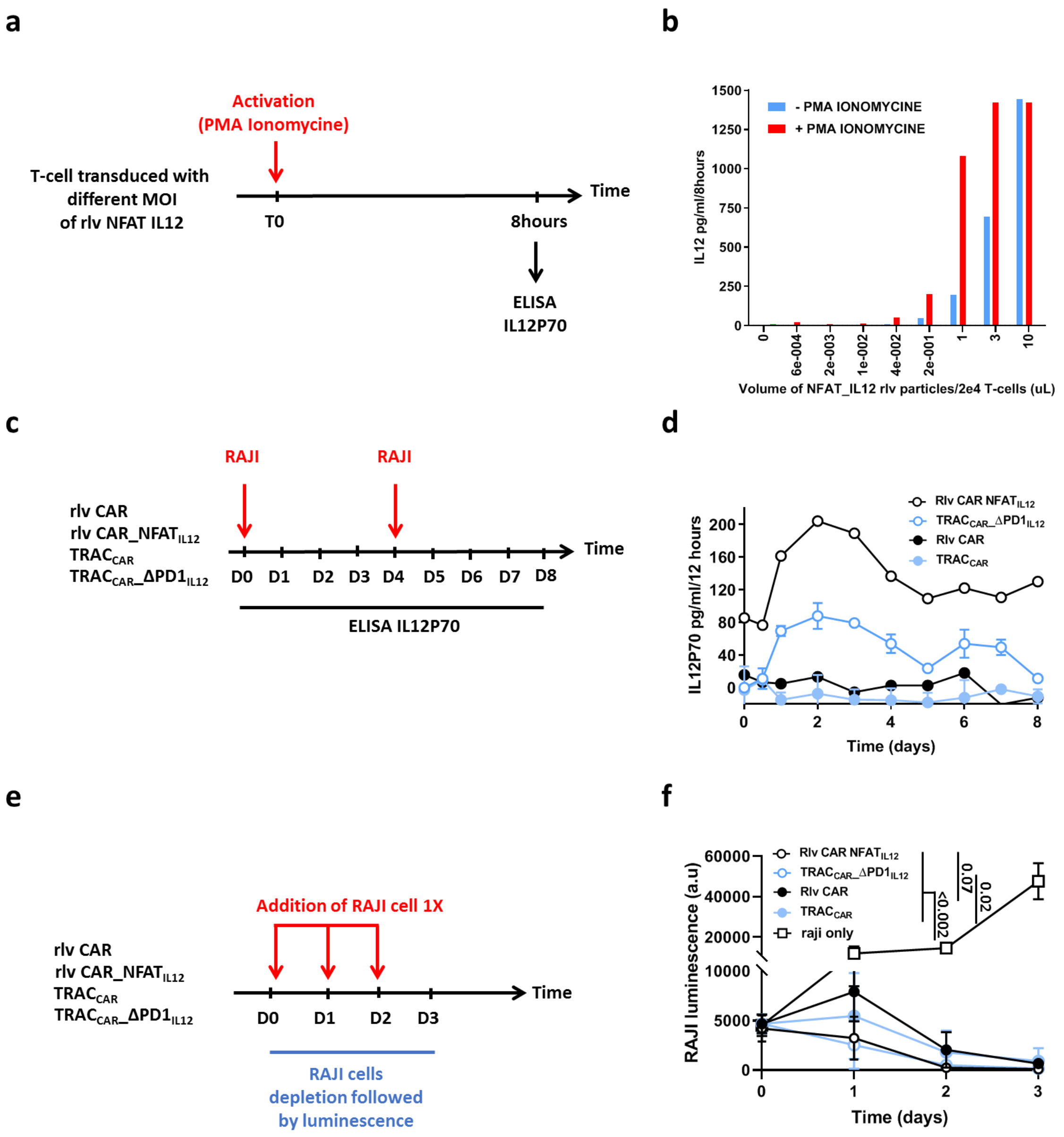


**Supplementary Figure 6. Functional comparison of AAV6- and rLV-based engineered T-cells.** Primary T-cells obtained from one donor were transduced by either $NFAT_{IL12}$ transgene-encoding lentiviral particles alone (different MOI), CAR transgene encoding lentiviral particles alone (MOI 5, [1]), or with a combination of both (MOI 5, [1] and MOI 0.25, [2, 3], respectively) to generate rLV T $NFAT_{IL12}$, rLV CAR and rLV CAR $NFAT_{IL12}$, respectively. $TRAC_{CAR}$ and $TRAC_{CAR}$_$\Delta PD1_{IL12}$ were produced according to the protocol described in Figure 1. **a** Schematic showing the experimental design used to investigate the basal and activation-dependent levels of IL-12P70 secretion by T $NFAT_{IL12}$. **b** Levels of IL-12P70 secretion by primary T-cells transduced by

increasing amounts of $NFAT_{IL12}$ transgene-encoding lentiviral particles, in the presence or absence of PMA Ionomycine (n=1). **c** Schematic showing the strategy used to assess the kinetic of IL-12P70 secretion by engineered $TRAC_{CAR}$ T-cells in the presence of tumor cells. **d** Kinetics of IL-12P70 secretion by engineered $TRAC_{CAR}$ T-cells. The data shown represent the average +/- standard deviation of two experiments performed with $TRAC_{CAR}$ and $TRAC_{CAR}$_ΔPD1$_{IL12}$ T-cells engineered from two different donors and one experiment performed with rLV CAR $NFAT_{IL12}$ engineered from a common donor. **e** Schematic showing the experimental design to investigate the long-term antitumor activity of engineered CAR T-cells. **f** Antitumor activity of the CAR T-cells monitored by RAJI tumor cells luminescence. Data represent the average of 4 experiments performed with $TRAC_{CAR}$, $TRAC_{CAR}$_ΔPD1$_{IL12}$ and rLV CAR T-cells engineered from 4 different donors and two experiments performed with rLV CAR $NFAT_{IL12}$ engineered from two common donors. The mean +/- standard deviation is represented. Significance of the difference between constructs was assessed using a linear mixed effects models, using the lme4 and lmerTest R packages, taking donor and days (1, 2 and 3) as random effects. p-value are indicated in the legend. Difference between $TRAC_{CAR}$/rLV CAR and $TRAC_{CAR}$ ΔPD1$_{IL12}$/rLV CAR $NFAT_{IL12}$ were found not significant. Source data are provided as a Source Data file.

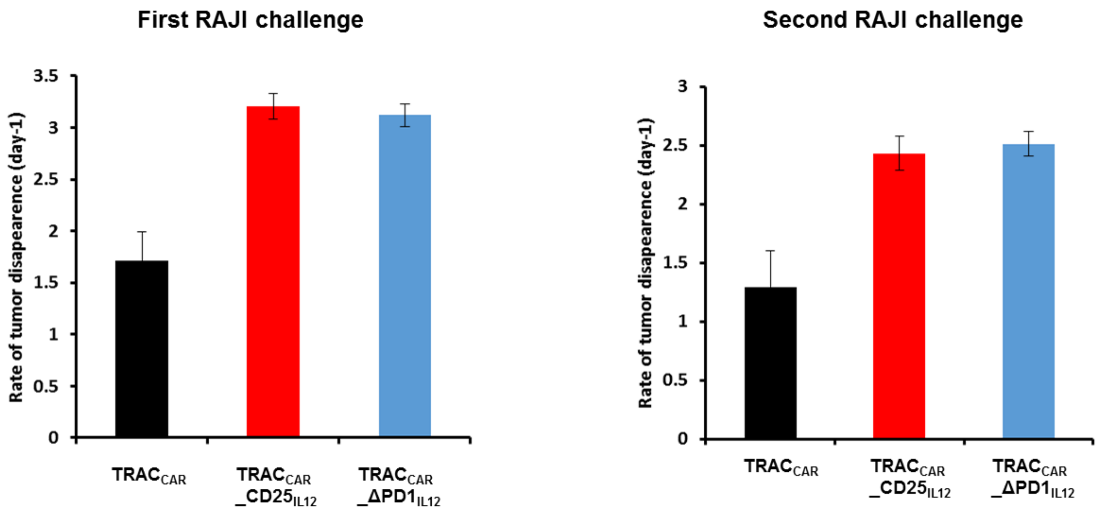


**Supplementary Figure 7. Kinetics of tumor cell disappearance *in vitro*.** $TRAC_{CAR}$ and $TRAC_{CAR}$_CD25$_{IL12}$ T-cells or $TRAC_{CAR}$ and $TRAC_{CAR}$_ΔPD1$_{IL12}$ T-cells were challenged with 1 equivalent of RAJI cells at Day 0 and Day 4 and cultivated for 8 days. The disappearance of the tumor cell was then analyzed as a function of time by analyzing flow cytometry data (using a CD19 antibody specific for RAJI cells) and the total number

of cells in the mixture. The kinetics of tumor cell disappearance was fitted to a decreasing monoexponential function. The rates of tumor cell disappearance obtained from the first (left) and second (right) challenge are plotted for each $TRAC_{CAR}$ T-cell group. The rate constants represent the average of two experiments performed with cells from 2 independent donors. The error bars show results +/- standard error. Source data are provided as a Source Data file.

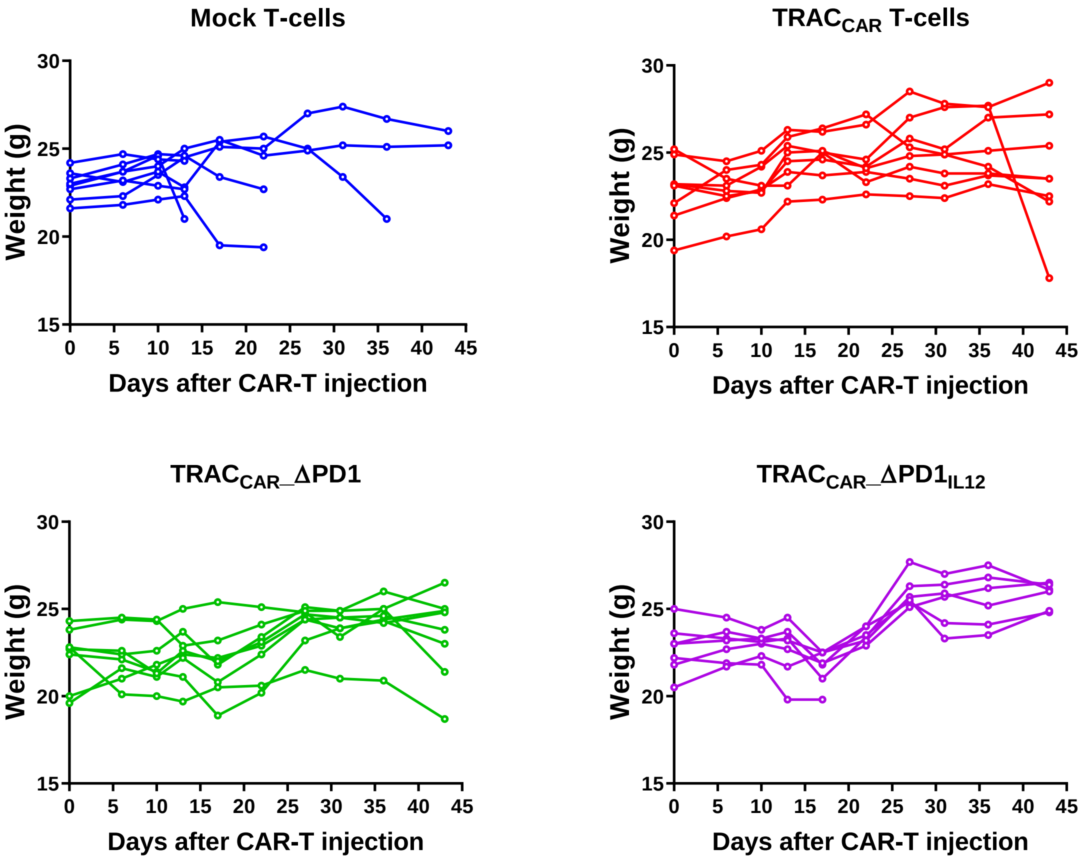


**Supplementary Figure 8. Influence of conditional secretion of IL-12P70 by $TRAC_{CAR}$ T-cells on mice weight.** Immunodeficient NSG mice were adoptively transferred on day 0 with RAJI-Luc-GFP tumor cells ($2.5 \times 10^5$ cells per animal in 100 µL of PBS i.v.). Tumor cells were allowed to expand until mice randomization, performed at day 3 on the basis of the level of tumor cell bioluminescence signal. On the same day, mice were adoptively transferred (i.v.) with $7\times10^6$ viable Mock-transduced T-cells (N=8), $TRAC_{CAR}$ T-cells (N=8), $TRAC_{CAR}$_ΔPD1 (N=8) or $TRAC_{CAR}$_ΔPD1$_{IL12}$ T-cells (N=7). The weight of individual mice was monitored on various days up to 43 days.

**Supplementary Table 1. Statistical analysis of $TRAC_{CAR}$ T-cell immunophenotyping.** The comparison of subpopulation frequencies documented in Fig. 7b was done using a linear mixed model, using lmer from the lme4 R package. P-value are indicated for each pair of groups considered at a given time (before and after 5 days of tumor-dependent activation of $TRAC_{CAR}$ T-cells).

| Day | Condition 1 | Condition 2 | Subpopulation | pValue |
|---|---|---|---|---|
| Day 0 | $TRAC_{CAR}$ _CD25 | $TRAC_{CAR}$ _$CD25_{IL12}$ | CD62L+ CD45RA+ | 0.658896 |
| Day 0 | $TRAC_{CAR}$ _CD25 | $TRAC_{CAR}$ _$CD25_{IL12}$ | CD62L+ CD45RA- | 0.184326 |
| Day 0 | $TRAC_{CAR}$ _CD25 | $TRAC_{CAR}$ _$CD25_{IL12}$ | CD62L- CD45RA+ | 0.000133 |
| Day 0 | $TRAC_{CAR}$ _CD25 | $TRAC_{CAR}$ _$CD25_{IL12}$ | CD62L- CD45RA- | 0.00012 |
| Day 0 | $TRAC_{CAR}$ _ΔPD1 | $TRAC_{CAR}$ _$ΔPD1_{IL12}$ | CD62L+ CD45RA+ | 0.036271 |
| Day 0 | $TRAC_{CAR}$ _ΔPD1 | $TRAC_{CAR}$ _$ΔPD1_{IL12}$ | CD62L+ CD45RA- | 0.011157 |
| Day 0 | $TRAC_{CAR}$ _ΔPD1 | $TRAC_{CAR}$ _$ΔPD1_{IL12}$ | CD62L- CD45RA+ | 0.002037 |
| Day 0 | $TRAC_{CAR}$ _ΔPD1 | $TRAC_{CAR}$ _$ΔPD1_{IL12}$ | CD62L- CD45RA- | 0.021477 |
| Day 0 | $TRAC_{CAR}$ | $TRAC_{CAR}$ _$ΔPD1_{IL12}$ | CD62L+ CD45RA+ | 0.634968 |
| Day 0 | $TRAC_{CAR}$ | $TRAC_{CAR}$ _$ΔPD1_{IL12}$ | CD62L+ CD45RA- | 0.581474 |
| Day 0 | $TRAC_{CAR}$ | $TRAC_{CAR}$ _$ΔPD1_{IL12}$ | CD62L- CD45RA+ | 0.644971 |
| Day 0 | $TRAC_{CAR}$ | $TRAC_{CAR}$ _$ΔPD1_{IL12}$ | CD62L- CD45RA- | 0.176879 |
| Day 0 | $TRAC_{CAR}$ | $TRAC_{CAR}$ _$CD25_{IL12}$ | CD62L+ CD45RA+ | 0.641691 |
| Day 0 | $TRAC_{CAR}$ | $TRAC_{CAR}$ _$CD25_{IL12}$ | CD62L+ CD45RA- | 0.347546 |
| Day 0 | $TRAC_{CAR}$ | $TRAC_{CAR}$ _$CD25_{IL12}$ | CD62L- CD45RA+ | 0.012708 |
| Day 0 | $TRAC_{CAR}$ | $TRAC_{CAR}$ _$CD25_{IL12}$ | CD62L- CD45RA- | 6.79E-06 |
| Day 5 | $TRAC_{CAR}$ _CD25 | $TRAC_{CAR}$ _$CD25_{IL12}$ | CD62L+ CD45RA+ | 0.367066 |
| Day 5 | $TRAC_{CAR}$ _CD25 | $TRAC_{CAR}$ _$CD25_{IL12}$ | CD62L+ CD45RA- | 0.079901 |
| Day 5 | $TRAC_{CAR}$ _CD25 | $TRAC_{CAR}$ _$CD25_{IL12}$ | CD62L- CD45RA+ | 0.063415 |
| Day 5 | $TRAC_{CAR}$ _CD25 | $TRAC_{CAR}$ _$CD25_{IL12}$ | CD62L- CD45RA- | 0.007554 |
| Day 5 | $TRAC_{CAR}$ _ΔPD1 | $TRAC_{CAR}$ _$ΔPD1_{IL12}$ | CD62L+ CD45RA+ | 0.084562 |
| Day 5 | $TRAC_{CAR}$ _ΔPD1 | $TRAC_{CAR}$ _$ΔPD1_{IL12}$ | CD62L+ CD45RA- | 0.935416 |
| Day 5 | $TRAC_{CAR}$ _ΔPD1 | $TRAC_{CAR}$ _$ΔPD1_{IL12}$ | CD62L- CD45RA+ | 0.053306 |
| Day 5 | $TRAC_{CAR}$ _ΔPD1 | $TRAC_{CAR}$ _$ΔPD1_{IL12}$ | CD62L- CD45RA- | 0.007324 |
| Day 5 | $TRAC_{CAR}$ | $TRAC_{CAR}$ _$ΔPD1_{IL12}$ | CD62L+ CD45RA+ | 0.09587 |
| Day 5 | $TRAC_{CAR}$ | $TRAC_{CAR}$ _$ΔPD1_{IL12}$ | CD62L+ CD45RA- | 0.659274 |
| Day 5 | $TRAC_{CAR}$ | $TRAC_{CAR}$ _$ΔPD1_{IL12}$ | CD62L- CD45RA+ | 0.020424 |
| Day 5 | $TRAC_{CAR}$ | $TRAC_{CAR}$ _$ΔPD1_{IL12}$ | CD62L- CD45RA- | 0.000118 |
| Day 5 | $TRAC_{CAR}$ | $TRAC_{CAR}$ _$CD25_{IL12}$ | CD62L+ CD45RA+ | 0.800903 |
| Day 5 | $TRAC_{CAR}$ | $TRAC_{CAR}$ _$CD25_{IL12}$ | CD62L+ CD45RA- | 0.000964 |
| Day 5 | $TRAC_{CAR}$ | $TRAC_{CAR}$ _$CD25_{IL12}$ | CD62L- CD45RA+ | 0.025266 |
| Day 5 | $TRAC_{CAR}$ | $TRAC_{CAR}$ _$CD25_{IL12}$ | CD62L- CD45RA- | 0.000654 |

**Supplementary Table 2. Statistical comparison of $TRAC_{CAR}$ T-cell antitumor activity in vivo.** The comparison of tumor growth was done using the aucVardiTest function of the clinfun R package[4]. P-value are indicated for each pair of groups considered

| Condition 1 | Condition 2 | pValue |
|---|---|---|
| Mock_T_cells | $TRAC_{CAR}$ | 0.00019996 |
| Mock_T_cells | $TRAC_{CAR}$ _ΔPD1 | 0.00019996 |
| Mock_T_cells | $TRAC_{CAR}$ _ $ΔPD1_{IL12}$ | 0.00039992 |
| $TRAC_{CAR}$ | $TRAC_{CAR}$ _ΔPD1 | 0.6628674 |
| $TRAC_{CAR}$ | $TRAC_{CAR}$ _$ΔPD1_{IL12}$ | 0.00119976 |
| $TRAC_{CAR}$ _PD1 | $TRAC_{CAR}$ _$ΔPD1_{IL12}$ | 0.0079984 |

## References


1. Valton, J. et al. A Versatile Safeguard for Chimeric Antigen Receptor T-Cell Immunotherapies. *Sci Rep* **8**, 8972 (2018).
2. Zhang, L. et al. Evaluation of γ-retroviral vectors that mediate the inducible expression of IL-12 for clinical application. *Journal of immuntherapy* **35**, 430-439 (2012).
3. Zhang, L. et al. Tumor-infiltrating lymphocytes genetically engineered with an inducible gene encoding interleukin-12 for the immunotherapy of metastatic melanoma. *Clin Cancer Res* **21**, 2278-2288 (2015).
4. Yehuda V, Z.Y.a.C.-H.Z. Two-Sample Tests for Growth Curves under Dependent Right Censoring *Biometrika* **88**, 949-960 (2001).
