# Supplementary material for: Repurposing endogenous immune pathways to tailor and control chimeric antigen receptor T cell functionality
Source: Nat Commun. 2019 Nov 13;10:5100. doi: 10.1038/s41467-019-13088-3 (PMC6853973; doi:10.1038/s41467-019-13088-3)
Supplement: Supplementary file 2 — Supplementary Data 1 [file 41467_2019_13088_MOESM2_ESM.docx]

**I-AAV6 repair matrices**

**-TRAC_CARm_**

The DNA sequence of TRAC_CARm_ is documented below. The subdomains embedded in this matrix are indicated using the following color code. ITR sequences, TRAC locus homology Left, HA Tag, T2A, Anti_CD22 ScFV, CD8 hinge, CD8 Transmembrane domain, 41BB, CD3 zeta, Stop codon, BGH PolyA, TRAC locus homology Right.

CCTGCAGGCAGCTGCGCGCTCGCTCGCTCACTGAGGCCGCCCGGGCAAAGCCCGGGCGTCGGGCGACCTTTGGTCGCCCGGCCTCAGTGAGCGAGCGAGCGCGCAGAGAGGGAGTGGCCAACTCCATCACTAGGGGTTCCTGCGGCCGCACGCGTAAGTAGCCCTGCATTTCAGGTTTCCTTGAGTGGCAGGCCAGGCCTGGCCGTGAACGTTCACTGAAATCATGGCCTCTTGGCCAAGATTGATAGCTTGTGCCTGTCCCTGAGTCCCAGTCCATCACGAGCAGCTGGTTTCTAAGATGCTATTTCCCGTATAAAGCATGAGACCGTGACTTGCCAGCCCCACAGAGCCCCGCCCTTGTCCATCACTGGCATCTGGACTCCAGCCTGGGTTGGGGCAAAGAGGGAAATGAGATCATGTCCTAACCCTGATCCTCTTGTCCCACAGATATCCAGTACCCCTACGACGTGCCCGACTACGCCTCCGGTGAGGGCAGAGGAAGTCTTCTAACATGCGGTGACGTGGAGGAGAATCCGGGCCCCGGATCCGCTCTGCCCGTCACCGCTCTGCTGCTGCCACTGGCACTGCTGCTGCACGCTGCTAGGCCCGGAGGGGGAGGCAGCTGCCCCTACAGCAACCCCAGCCTGTGCAGCGGAGGCGGCGGCAGCGGCGGAGGGGGTAGCCAGGTGCAGCTGCAGCAGAGCGGCCCTGGCCTGGTGAAGCCAAGCCAGACACTGTCCCTGACCTGCGCCATCAGCGGCGATTCCGTGAGCTCCAACTCCGCCGCCTGGAATTGGATCAGGCAGTCCCCTTCTCGGGGCCTGGAGTGGCTGGGAAGGACATACTATCGGTCTAAGTGGTACAACGATTATGCCGTGTCTGTGAAGAGCAGAATCACAATCAACCCTGACACCTCCAAGAATCAGTTCTCTCTGCAGCTGAATAGCGTGACACCAGAGGACACCGCCGTGTACTATTGCGCCAGGGAGGTGACCGGCGACCTGGAGGATGCCTTTGACATCTGGGGCCAGGGCACAATGGTGACCGTGAGCTCCGGAGGCGGCGGATCTGGCGGAGGAGGAAGTGGGGGCGGCGGGAGTGATATCCAGATGACACAGTCCCCATCCTCTCTGAGCGCCTCCGTGGGCGACAGAGTGACAATCACCTGTAGGGCCTCCCAGACCATCTGGTCTTACCTGAACTGGTATCAGCAGAGGCCCGGCAAGGCCCCTAATCTGCTGATCTACGCAGCAAGCTCCCTGCAGAGCGGAGTGCCATCCAGATTCTCTGGCAGGGGCTCCGGCACAGACTTCACCCTGACCATCTCTAGCCTGCAGGCCGAGGACTTCGCCACCTACTATTGCCAGCAGTCTTATAGCATCCCCCAGACATTTGGCCAGGGCACCAAGCTGGAGATCAAGTCGGATCCCGGAAGCGGAGGGGGAGGCAGCTGCCCCTACAGCAACCCCAGCCTGTGCAGCGGAGGCGGCGGCAGCGAGCTGCCCACCCAGGGCACCTTCTCCAACGTGTCCACCAACGTGAGCCCAGCCAAGCCCACCACCACCGCCTGTCCTTATTCCAATCCTTCCCTGTGTGCTCCCACCACAACCCCCGCTCCAAGGCCCCCTACCCCCGCACCAACTATTGCCTCCCAGCCACTCTCACTGCGGCCTGAGGCCTGTCGGCCCGCTGCTGGAGGCGCAGTGCATACAAGGGGCCTCGATTTCGCCTGCGATATTTACATCTGGGCACCCCTCGCCGGCACCTGCGGGGTGCTTCTCCTCTCCCTGGTGATTACCCTGTATTGCAGACGGGGCCGGAAGAAGCTCCTCTACATTTTTAAGCAGCCTTTCATGCGGCCAGTGCAGACAACCCAAGAGGAGGATGGGTGTTCCTGCAGATTCCCTGAGGAAGAGGAAGGCGGGTGCGAGCTGAGAGTGAAGTTCTCCAGGAGCGCAGATGCCCCCGCCTATCAACAGGGCCAGAACCAGCTCTACAACGAGCTTAACCTCGGGAGGCGCGAAGAATACGACGTGTTGGATAAGAGAAGGGGGCGGGACCCCGAGATGGGAGGAAAGCCCCGGAGGAAGAACCCTCAGGAGGGCCTGTACAACGAGCTGCAGAAGGATAAGATGGCCGAGGCCTACTCAGAGATCGGGATGAAGGGGGAGCGGCGCCGCGGGAAGGGGCACGATGGGCTCTACCAGGGGCTGAGCACAGCCACAAAGGACACATACGACGCCTTGCACATGCAGGCCCTTCCACCCCGGGAATAGTCTAGAGGGCCCGTTTAAACCCGCTGATCAGCCTCGACTGTGCCTTCTAGTTGCCAGCCATCTGTTGTTTGCCCCTCCCCCGTGCCTTCCTTGACCCTGGAAGGTGCCACTCCCACTGTCCTTTCCTAATAAAATGAGGAAATTGCATCGCATTGTCTGAGTAGGTGTCATTCTATTCTGGGGGGTGGGGTGGGGCAGGACAGCAAGGGGGAGGATTGGGAAGACAATAGCAGGCATGCTGGGGATGCGGTGGGCTCTATGACTAGTGGCGAATTCCCGTGTACCAGCTGAGAGACTCTAAATCCAGTGACAAGTCTGTCTGCCTATTCACCGATTTTGATTCTCAAACAAATGTGTCACAAAGTAAGGATTCTGATGTGTATATCACAGACAAAACTGTGCTAGACATGAGGTCTATGGACTTCAAGAGCAACAGTGCTGTGGCCTGGAGCAACAAATCTGACTTTGCATGTGCAAACGCCTTCAACAACAGCATTATTCCAGAAGACACCTTCTTCCCCAGCCCAGGTAAGGGCAGCTTTGGTGCCTTCGCAGGCTGTTTCCTTGCTTCAGGAAACCGGACCACGTGCGGACCGAGCGGCCGCAGGAACCCCTAGTGATGGAGTTGGCCACTCCCTCTCTGCGCGCTCGCTCGCTCACTGAGGCCGGGCGACCAAAGGTCGCCCGACGCCCGGGCTTTGCCCGGGCGGCCTCAGTGAGCGAGCGAGCGCGCAGCTGCCTGCAGG

**-PDCD1_IL12m_**

The DNA sequence of PDCD1_IL12m_ is documented below. The subdomains embedded in this matrix are indicated using the following color code. ITR sequences, PD1 locus homology Left, F2A, IL-12 alpha subunit, P2A, IL-12 beta subunit, T2A, ΔLNGFR, Stop codon, BGH PolyA, PD1 locus homology Right.

CCTGCAGGCAGCTGCGCGCTCGCTCGCTCACTGAGGCCGCCCGGGCAAAGCCCGGGCGTCGGGCGACCTTTGGTCGCCCGGCCTCAGTGAGCGAGCGAGCGCGCAGAGAGGGAGTGGCCAACTCCATCACTAGGGGTTCCTGCGGCCGCACGCGTACTAGTGACTCCCCAGACAGGCCCTGGAACCCCCCCACCTTCTCCCCAGCCCTGCTCGTGGTGACCGAAGGGGACAACGCCACCTTCACCTGCAGCTTCTCCAACACATCGGAGAGCTTCGTGCTAAACTGGTACCGCATGAGCCCCAGCAACCAGACGGACAAGCTGGCCGCCTTCCCCGAGGACCGCAGCCAGCCCGGCCAGGACTGCCGCTTCCGTGTCACACAACTGCCCAACGGGCGTGACTTCCACATGAGCGTGGTCAGGGCCCGGCGCAATGACAGCGGCACCTACCTCTGTGGGGCCGGTTCTGGCGTGAAACAGACTTTGAATTTTGACCTTCTCAAGTTGGCGGGAGACGTGGAGTCCAACCCAGGGCCCATGTGGCCCCCTGGGTCAGCCTCCCAGCCACCGCCCTCACCTGCCGCGGCCACAGGTCTGCATCCAGCGGCTCGCCCTGTGTCCCTGCAGTGCCGGCTCAGCATGTGTCCAGCGCGCAGCCTCCTCCTTGTGGCTACCCTGGTCCTCCTGGACCACCTCAGTTTGGCCAGAAACCTCCCCGTGGCCACTCCAGACCCAGGAATGTTCCCATGCCTTCACCACTCCCAAAACCTGCTGAGGGCCGTCAGCAACATGCTCCAGAAGGCCAGACAAACTCTAGAATTTTACCCTTGCACTTCTGAAGAGATTGATCATGAAGATATCACAAAAGATAAAACCAGCACAGTGGAGGCCTGTTTACCATTGGAATTAACCAAGAATGAGAGTTGCCTAAATTCCAGAGAGACCTCTTTCATAACTAATGGGAGTTGCCTGGCCTCCAGAAAGACCTCTTTTATGATGGCCCTGTGCCTTAGTAGTATTTATGAAGACTTGAAGATGTACCAGGTGGAGTTCAAGACCATGAATGCAAAGCTTCTGATGGATCCTAAGAGGCAGATCTTTCTAGATCAAAACATGCTGGCAGTTATTGATGAGCTGATGCAGGCCCTGAATTTCAACAGTGAGACTGTGCCACAAAAATCCTCCCTTGAAGAACCGGATTTTTATAAAACTAAAATCAAGCTCTGCATACTTCTTCATGCTTTCAGAATTCGGGCAGTGACTATTGATAGAGTGATGAGCTATCTGAATGCTTCCGGAAGCGGAGCTACTAACTTCAGCCTGCTGAAGCAGGCTGGAGACGTGGAGGAGAACCCTGGACCTATGTGTCACCAGCAGTTGGTCATCTCTTGGTTTTCCCTGGTTTTTCTGGCATCTCCCCTCGTGGCCATATGGGAACTGAAGAAAGATGTTTATGTCGTAGAATTGGATTGGTATCCGGATGCCCCTGGAGAAATGGTGGTCCTCACCTGTGACACCCCTGAAGAAGATGGTATCACCTGGACCTTGGACCAGAGCAGTGAGGTCTTAGGCTCTGGCAAAACCCTGACCATCCAAGTCAAAGAGTTTGGAGATGCTGGCCAGTACACCTGTCACAAAGGAGGCGAGGTTCTAAGCCATTCGCTCCTGCTGCTTCACAAAAAGGAAGATGGAATTTGGTCCACTGATATTTTAAAGGACCAGAAAGAACCCAAAAATAAGACCTTTCTAAGATGCGAGGCCAAGAATTATTCTGGACGTTTCACCTGCTGGTGGCTGACGACAATCAGTACTGATTTGACATTCAGTGTCAAAAGCAGCAGAGGCTCTTCTGACCCCCAAGGGGTGACGTGCGGAGCTGCTACACTCTCTGCAGAGAGAGTCAGAGGGGACAACAAGGAGTATGAGTACTCAGTGGAGTGCCAGGAGGACAGTGCCTGCCCAGCTGCTGAGGAGAGTCTGCCCATTGAGGTCATGGTGGATGCCGTTCACAAGCTCAAGTATGAAAACTACACCAGCAGCTTCTTCATCAGGGACATCATCAAACCTGACCCACCCAAGAACTTGCAGCTGAAGCCATTAAAGAATTCTCGGCAGGTGGAGGTCAGCTGGGAGTACCCTGACACCTGGAGTACTCCACATTCCTACTTCTCCCTGACATTCTGCGTTCAGGTCCAGGGCAAGAGCAAGAGAGAAAAGAAAGATAGAGTCTTCACGGACAAGACCTCAGCCACGGTCATCTGCCGCAAAAATGCCAGCATTAGCGTGCGGGCCCAGGACCGCTACTATAGCTCATCTTGGAGCGAATGGGCATCTGTGCCCTGCAGTGAGGGCAGAGGCAGCCTGCTGACCTGCGGCGACGTCGAGGAGAACCCCGGGCCCATGGGGGCAGGTGCCACCGGCCGCGCCATGGACGGGCCGCGCCTGCTGCTGTTGCTGCTTCTGGGGGTGTCCCTTGGAGGTGCCAAGGAGGCATGCCCCACAGGCCTGTACACACACAGCGGTGAGTGCTGCAAAGCCTGCAACCTGGGCGAGGGTGTGGCCCAGCCTTGTGGAGCCAACCAGACCGTGTGTGAGCCCTGCCTGGACAGCGTGACGTTCTCCGACGTGGTGAGCGCGACCGAGCCGTGCAAGCCGTGCACCGAGTGCGTGGGGCTCCAGAGCATGTCGGCGCCGTGCGTGGAGGCCGATGACGCCGTGTGCCGCTGCGCCTACGGCTACTACCAGGATGAGACGACTGGGCGCTGCGAGGCGTGCCGCGTGTGCGAGGCGGGCTCGGGCCTCGTGTTCTCCTGCCAGGACAAGCAGAACACCGTGTGCGAGGAGTGCCCCGACGGCACGTATTCCGACGAGGCCAACCACGTGGACCCGTGCCTGCCCTGCACCGTGTGCGAGGACACCGAGCGCCAGCTCCGCGAGTGCACACGCTGGGCCGACGCCGAGTGCGAGGAGATCCCTGGCCGTTGGATTACACGGTCCACACCCCCAGAGGGCTCGGACAGCACAGCCCCCAGCACCCAGGAGCCTGAGGCACCTCCAGAACAAGACCTCATAGCCAGCACGGTGGCAGGTGTGGTGACCACAGTGATGGGCAGCTCCCAGCCCGTGGTGACCCGAGGCACCACCGACAACCTCATCCCTGTCTATTGCTCCATCCTGGCTGCTGTGGTTGTGGGTCTTGTGGCCTACATAGCCTTCAAGAGGTGATCTAGAGGGCCCGTTTAAACCCGCTGATCAGCCTCGACTGTGCCTTCTAGTTGCCAGCCATCTGTTGTTTGCCCCTCCCCCGTGCCTTCCTTGACCCTGGAAGGTGCCACTCCCACTGTCCTTTCCTAATAAAATGAGGAAATTGCATCGCATTGTCTGAGTAGGTGTCATTCTATTCTGGGGGGTGGGGTGGGGCAGGACAGCAAGGGGGAGGATTGGGAAGACAATAGCAGGCATGCTGGGGATGCGGTGGGCTCTATGACTAGTGGCGAATTCGGCGCAGATCAAAGAGAGCCTGCGGGCAGAGCTCAGGGTGACAGGTGCGGCCTCGGAGGCCCCGGGGCAGGGGTGAGCTGAGCCGGTCCTGGGGTGGGTGTCCCCTCCTGCACAGGATCAGGAGCTCCAGGGTCGTAGGGCAGGGACCCCCCAGCTCCAGTCCAGGGCTCTGTCCTGCACCTGGGGAATGGTGACCGGCATCTCTGTCCTCTAGCTCTGGAAGCACCCCAGCCCCTCTAGTCTGCCCTCACCCCTGACCCTGACCCTCCACCCTGACCCCGTCCTAACCCCTGACCTTTGTCCGGACCACGTGCGGACCGAGCGGCCGCAGGAACCCCTAGTGATGGAGTTGGCCACTCCCTCTCTGCGCGCTCGCTCGCTCACTGAGGCCGGGCGACCAAAGGTCGCCCGACGCCCGGGCTTTGCCCGGGCGGCCTCAGTGAGCGAGCGAGCGCGCAGCTGCCTGCAGG

**-IL2rα_IL12m_**

The DNA sequence of IL2rα_IL12m_ is documented below. The subdomains embedded in this matrix are indicated using the following color code**.** ITR sequences, IL2rα locus homology Left, F2A, IL-12 alpha subunit, P2A, IL-12 beta subunit, T2A, ΔLNGFR, Stop codon, IL2rα locus homology Right.

CCTGCAGGCAGCTGCGCGCTCGCTCGCTCACTGAGGCCGCCCGGGCAAAGCCCGGGCGTCGGGCGACCTTTGGTCGCCCGGCCTCAGTGAGCGAGCGAGCGCGCAGAGAGGGAGTGGCCAACTCCATCACTAGGGGTTCCTGCGGCCGCACGCGTACTAGTGTTTATTATTCCTGTTCCACAGCTATTGTCTGCCATATAAAAACTTAGGCCAGGCACAGTGGCTCACACCTGTAATCCCAGCACTTTGGAAGGCCGAGGCAGGCAGATCACAAGGTCAGGAGTTCGAGACCAGCCTGGCCAACATAGCAAAACCCCATCTCTACTAAAAATACAAAAATTAGCCAGGCATGGTGGCGTGTGCACTGGTTTAGAGTGAGGACCACATTTTTTTGGTGCCGTGTTACACATATGACCGTGACTTTGTTACACCACTACAGGAGGAAGAGTAGAAGAACAATCGGTTCTGGCGTGAAACAGACTTTGAATTTTGACCTTCTCAAGTTGGCGGGAGACGTGGAGTCCAACCCAGGGCCCATGTGGCCCCCTGGGTCAGCCTCCCAGCCACCGCCCTCACCTGCCGCGGCCACAGGTCTGCATCCAGCGGCTCGCCCTGTGTCCCTGCAGTGCCGGCTCAGCATGTGTCCAGCGCGCAGCCTCCTCCTTGTGGCTACCCTGGTCCTCCTGGACCACCTCAGTTTGGCCAGAAACCTCCCCGTGGCCACTCCAGACCCAGGAATGTTCCCATGCCTTCACCACTCCCAAAACCTGCTGAGGGCCGTCAGCAACATGCTCCAGAAGGCCAGACAAACTCTAGAATTTTACCCTTGCACTTCTGAAGAGATTGATCATGAAGATATCACAAAAGATAAAACCAGCACAGTGGAGGCCTGTTTACCATTGGAATTAACCAAGAATGAGAGTTGCCTAAATTCCAGAGAGACCTCTTTCATAACTAATGGGAGTTGCCTGGCCTCCAGAAAGACCTCTTTTATGATGGCCCTGTGCCTTAGTAGTATTTATGAAGACTTGAAGATGTACCAGGTGGAGTTCAAGACCATGAATGCAAAGCTTCTGATGGATCCTAAGAGGCAGATCTTTCTAGATCAAAACATGCTGGCAGTTATTGATGAGCTGATGCAGGCCCTGAATTTCAACAGTGAGACTGTGCCACAAAAATCCTCCCTTGAAGAACCGGATTTTTATAAAACTAAAATCAAGCTCTGCATACTTCTTCATGCTTTCAGAATTCGGGCAGTGACTATTGATAGAGTGATGAGCTATCTGAATGCTTCCGGAAGCGGAGCTACTAACTTCAGCCTGCTGAAGCAGGCTGGAGACGTGGAGGAGAACCCTGGACCTATGTGTCACCAGCAGTTGGTCATCTCTTGGTTTTCCCTGGTTTTTCTGGCATCTCCCCTCGTGGCCATATGGGAACTGAAGAAAGATGTTTATGTCGTAGAATTGGATTGGTATCCGGATGCCCCTGGAGAAATGGTGGTCCTCACCTGTGACACCCCTGAAGAAGATGGTATCACCTGGACCTTGGACCAGAGCAGTGAGGTCTTAGGCTCTGGCAAAACCCTGACCATCCAAGTCAAAGAGTTTGGAGATGCTGGCCAGTACACCTGTCACAAAGGAGGCGAGGTTCTAAGCCATTCGCTCCTGCTGCTTCACAAAAAGGAAGATGGAATTTGGTCCACTGATATTTTAAAGGACCAGAAAGAACCCAAAAATAAGACCTTTCTAAGATGCGAGGCCAAGAATTATTCTGGACGTTTCACCTGCTGGTGGCTGACGACAATCAGTACTGATTTGACATTCAGTGTCAAAAGCAGCAGAGGCTCTTCTGACCCCCAAGGGGTGACGTGCGGAGCTGCTACACTCTCTGCAGAGAGAGTCAGAGGGGACAACAAGGAGTATGAGTACTCAGTGGAGTGCCAGGAGGACAGTGCCTGCCCAGCTGCTGAGGAGAGTCTGCCCATTGAGGTCATGGTGGATGCCGTTCACAAGCTCAAGTATGAAAACTACACCAGCAGCTTCTTCATCAGGGACATCATCAAACCTGACCCACCCAAGAACTTGCAGCTGAAGCCATTAAAGAATTCTCGGCAGGTGGAGGTCAGCTGGGAGTACCCTGACACCTGGAGTACTCCACATTCCTACTTCTCCCTGACATTCTGCGTTCAGGTCCAGGGCAAGAGCAAGAGAGAAAAGAAAGATAGAGTCTTCACGGACAAGACCTCAGCCACGGTCATCTGCCGCAAAAATGCCAGCATTAGCGTGCGGGCCCAGGACCGCTACTATAGCTCATCTTGGAGCGAATGGGCATCTGTGCCCTGCAGTGAGGGCAGAGGCAGCCTGCTGACCTGCGGCGACGTCGAGGAGAACCCCGGGCCCATGGGGGCAGGTGCCACCGGCCGCGCCATGGACGGGCCGCGCCTGCTGCTGTTGCTGCTTCTGGGGGTGTCCCTTGGAGGTGCCAAGGAGGCATGCCCCACAGGCCTGTACACACACAGCGGTGAGTGCTGCAAAGCCTGCAACCTGGGCGAGGGTGTGGCCCAGCCTTGTGGAGCCAACCAGACCGTGTGTGAGCCCTGCCTGGACAGCGTGACGTTCTCCGACGTGGTGAGCGCGACCGAGCCGTGCAAGCCGTGCACCGAGTGCGTGGGGCTCCAGAGCATGTCGGCGCCGTGCGTGGAGGCCGATGACGCCGTGTGCCGCTGCGCCTACGGCTACTACCAGGATGAGACGACTGGGCGCTGCGAGGCGTGCCGCGTGTGCGAGGCGGGCTCGGGCCTCGTGTTCTCCTGCCAGGACAAGCAGAACACCGTGTGCGAGGAGTGCCCCGACGGCACGTATTCCGACGAGGCCAACCACGTGGACCCGTGCCTGCCCTGCACCGTGTGCGAGGACACCGAGCGCCAGCTCCGCGAGTGCACACGCTGGGCCGACGCCGAGTGCGAGGAGATCCCTGGCCGTTGGATTACACGGTCCACACCCCCAGAGGGCTCGGACAGCACAGCCCCCAGCACCCAGGAGCCTGAGGCACCTCCAGAACAAGACCTCATAGCCAGCACGGTGGCAGGTGTGGTGACCACAGTGATGGGCAGCTCCCAGCCCGTGGTGACCCGAGGCACCACCGACAACCTCATCCCTGTCTATTGCTCCATCCTGGCTGCTGTGGTTGTGGGTCTTGTGGCCTACATAGCCTTCAAGAGGTGAAAAACCAAAAGAACAAGAATTTCTTGGTAAGAAGCCGGGAACAGACAACAGAAGTCATGAAGCCCAAGTGAAATCAAAGGTGCTAAATGGTCGCCCAGGAGACATCCGTTGTGCTTGCCTGCGTTTTGGAAGCTCTGAAGTCACATCACAGGACACGGGGCAGTGGCAACCTTGTCTCTATGCCAGCTCAGTCCCATCAGAGAGCGAGCGCTACCCACTTCTAAATAGCAATTTCGCCGTTGAAGAGGAAGGGCAAAACCACTAGAACTCTCCATCTTATTTTCATGTATATGTGTTCATTCCGGACCACGTGCGGACCGAGCGGCCGCAGGAACCCCTAGTGATGGAGTTGGCCACTCCCTCTCTGCGCGCTCGCTCGCTCACTGAGGCCGGGCGACCAAAGGTCGCCCGACGCCCGGGCTTTGCCCGGGCGGCCTCAGTGAGCGAGCGAGCGCGCAGCTGCCTGCAGG

**II-TALEN amino acid sequences**

**-TRAC TALEN LEFT arm**

The aminoacid sequence of TRAC TALEN LEFT arm is documented below

MGDPKKKRKVIDIADLRTLGYSQQQQEKIKPKVRSTVAQHHEALVGHGFTHAHIVALSQHPAALGTVAVKYQDMIAALPEATHEAIVGVGKQWSGARALEALLTVAGELRGPPLQLDTGQLLKIAKRGGVTAVEAVHAWRNALTGAPLNLTPQQVVAIASNGGGKQALETVQRLLPVLCQAHGLTPQQVVAIASNNGGKQALETVQRLLPVLCQAHGLTPQQVVAIASNGGGKQALETVQRLLPVLCQAHGLTPEQVVAIASHDGGKQALETVQRLLPVLCQAHGLTPEQVVAIASHDGGKQALETVQRLLPVLCQAHGLTPEQVVAIASHDGGKQALETVQRLLPVLCQAHGLTPEQVVAIASNIGGKQALETVQALLPVLCQAHGLTPEQVVAIASHDGGKQALETVQRLLPVLCQAHGLTPEQVVAIASNIGGKQALETVQALLPVLCQAHGLTPQQVVAIASNNGGKQALETVQRLLPVLCQAHGLTPEQVVAIASNIGGKQALETVQALLPVLCQAHGLTPQQVVAIASNGGGKQALETVQRLLPVLCQAHGLTPEQVVAIASNIGGKQALETVQALLPVLCQAHGLTPQQVVAIASNGGGKQALETVQRLLPVLCQAHGLTPEQVVAIASHDGGKQALETVQRLLPVLCQAHGLTPQQVVAIASNGGGRPALESIVAQLSRPDPALAALTNDHLVALACLGGRPALDAVKKGLGDPISRSQLVKSELEEKKSELRHKLKYVPHEYIELIEIARNSTQDRILEMKVMEFFMKVYGYRGKHLGGSRKPDGAIYTVGSPIDYGVIVDTKAYSGGYNLPIGQADEMQRYVEENQTRNKHINPNEWWKVYPSSVTEFKFLFVSGHFKGNYKAQLTRLNHITNCNGAVLSVEELLIGGEMIKAGTLTLEEVRRKFNNGEINFAAD

**-TRAC TALEN RIGHT arm**

The aminoacid sequence of TRAC TALEN RIGHT arm is documented below

MGDPKKKRKVIDIADLRTLGYSQQQQEKIKPKVRSTVAQHHEALVGHGFTHAHIVALSQHPAALGTVAVKYQDMIAALPEATHEAIVGVGKQWSGARALEALLTVAGELRGPPLQLDTGQLLKIAKRGGVTAVEAVHAWRNALTGAPLNLTPEQVVAIASHDGGKQALETVQRLLPVLCQAHGLTPQQVVAIASNGGGKQALETVQRLLPVLCQAHGLTPEQVVAIASHDGGKQALETVQRLLPVLCQAHGLTPEQVVAIASNIGGKQALETVQALLPVLCQAHGLTPQQVVAIASNNGGKQALETVQRLLPVLCQAHGLTPEQVVAIASHDGGKQALETVQRLLPVLCQAHGLTPQQVVAIASNGGGKQALETVQRLLPVLCQAHGLTPQQVVAIASNNGGKQALETVQRLLPVLCQAHGLTPQQVVAIASNNGGKQALETVQRLLPVLCQAHGLTPQQVVAIASNGGGKQALETVQRLLPVLCQAHGLTPEQVVAIASNIGGKQALETVQALLPVLCQAHGLTPEQVVAIASHDGGKQALETVQRLLPVLCQAHGLTPEQVVAIASNIGGKQALETVQALLPVLCQAHGLTPEQVVAIASHDGGKQALETVQRLLPVLCQAHGLTPQQVVAIASNNGGKQALETVQRLLPVLCQAHGLTPQQVVAIASNGGGRPALESIVAQLSRPDPALAALTNDHLVALACLGGRPALDAVKKGLGDPISRSQLVKSELEEKKSELRHKLKYVPHEYIELIEIARNSTQDRILEMKVMEFFMKVYGYRGKHLGGSRKPDGAIYTVGSPIDYGVIVDTKAYSGGYNLPIGQADEMQRYVEENQTRNKHINPNEWWKVYPSSVTEFKFLFVSGHFKGNYKAQLTRLNHITNCNGAVLSVEELLIGGEMIKAGTLTLEEVRRKFNNGEINFAAD

**-PD1 TALEN LEFT arm**

The aminoacid sequence of PD1 TALEN LEFT arm is documented below

MGDPKKKRKVIDYPYDVPDYAIDIADLRTLGYSQQQQEKIKPKVRSTVAQHHEALVGHGFTHAHIVALSQHPAALGTVAVKYQDMIAALPEATHEAIVGVGKQWSGARALEALLTVAGELRGPPLQLDTGQLLKIAKRGGVTAVEAVHAWRNALTGAPLNLTPEQVVAIASKLGGKQALETVQALLPVLCQAHGLTPEQVVAIASHDGGKQALETVQRLLPVLCQAHGLTPEQVVAIASHDGGKQALETVQRLLPVLCQAHGLTPQQVVAIASNGGGKQALETVQRLLPVLCQAHGLTPEQVVAIASHDGGKQALETVQRLLPVLCQAHGLTPQQVVAIASNGGGKQALETVQRLLPVLCQAHGLTPQQVVAIASYKGGKQALETVQRLLPVLCQAHGLTPQQVVAIASNGGGKQALETVQRLLPVLCQAHGLTPQQVVAIASNNGGKQALETVQRLLPVLCQAHGLTPQQVVAIASNNGGKQALETVQRLLPVLCQAHGLTPQQVVAIASNNGGKQALETVQRLLPVLCQAHGLTPQQVVAIASNNGGKQALETVQRLLPVLCQAHGLTPEQVVAIASHDGGKQALETVQRLLPVLCQAHGLTPEQVVAIASHDGGKQALETVQRLLPVLCQAHGLTPEQVVAIASNIGGKQALETVQALLPVLCQAHGLTPQQVVAIASNGGGRPALESIVAQLSRPDPALAALTNDHLVALACLGGRPALDAVKKGLGDPISRSQLVKSELEEKKSELRHKLKYVPHEYIELIEIARNSTQDRILEMKVMEFFMKVYGYRGKHLGGSRKPDGAIYTVGSPIDYGVIVDTKAYSGGYNLPIGQADEMQRYVEENQTRNKHINPNEWWKVYPSSVTEFKFLFVSGHFKGNYKAQLTRLNHITNCNGAVLSVEELLIGGEMIKAGTLTLEEVRRKFNNGEINFAAD

**-PD1 TALEN RIGHT arm**

The aminoacid sequence of PD1 TALEN RIGHT arm is documented below

MGDPKKKRKVIDKETAAAKFERQHMDSIDIADLRTLGYSQQQQEKIKPKVRSTVAQHHEALVGHGFTHAHIVALSQHPAALGTVAVKYQDMIAALPEATHEAIVGVGKQWSGARALEALLTVAGELRGPPLQLDTGQLLKIAKRGGVTAVEAVHAWRNALTGAPLNLTPEQVVAIASHDGGKQALETVQRLLPVLCQAHGLTPQQVVAIASNGGGKQALETVQRLLPVLCQAHGLTPEQVVAIASHDGGKQALETVQRLLPVLCQAHGLTPQQVVAIASNGGGKQALETVQRLLPVLCQAHGLTPQQVVAIASNGGGKQALETVQRLLPVLCQAHGLTPQQVVAIASNGGGKQALETVQRLLPVLCQAHGLTPQQVVAIASNNGGKQALETVQRLLPVLCQAHGLTPEQVVAIASNIGGKQALETVQALLPVLCQAHGLTPQQVVAIASNGGGKQALETVQRLLPVLCQAHGLTPEQVVAIASHDGGKQALETVQRLLPVLCQAHGLTPQQVVAIASNGGGKQALETVQRLLPVLCQAHGLTPQQVVAIASNNGGKQALETVQRLLPVLCQAHGLTPEQVVAIASNGGKQALETVQRLLPVLCQAHGLTPQQVVAIASNNGGKQALETVQRLLPVLCQAHGLTPEQVVAIASHDGGKQALETVQRLLPVLCQAHGLTPQQVVAIASNGGGRPALESIVAQLSRPDPALAALTNDHLVALACLGGRPALDAVKKGLGDPISRSQLVKSELEEKKSELRHKLKYVPHEYIELIEIARNSTQDRILEMKVMEFFMKVYGYRGKHLGGSRKPDGAIYTVGSPIDYGVIVDTKAYSGGYNLPIGQADEMQRYVEENQTRNKHINPNEWWKVYPSSVTEFKFLFVSGHFKGNYKAQLTRLNHITNCNGAVLSVEELLIGGEMIKAGTLTLEEVRRKFNNGEINFAAD

**-IL2Rα TALEN LEFT arm**

The aminoacid sequence of IL2Rα TALEN LEFT arm is documented below

MGDPKKKRKVIDYPYDVPDYAIDIADLRTLGYSQQQQEKIKPKVRSTVAQHHEALVGHGFTHAHIVALSQHPAALGTVAVKYQDMIAALPEATHEAIVGVGKQWSGARALEALLTVAGELRGPPLQLDTGQLLKIAKRGGVTAVEAVHAWRNALTGAPLNLTPEQVVAIASNIGGKQALETVQALLPVLCQAHGLTPEQVVAIASHDGGKQALETVQRLLPVLCQAHGLTPEQVVAIASNIGGKQALETVQALLPVLCQAHGLTPQQVVAIASNNGGKQALETVQRLLPVLCQAHGLTPQQVVAIASNNGGKQALETVQRLLPVLCQAHGLTPEQVVAIASNIGGKQALETVQALLPVLCQAHGLTPQQVVAIASNNGGKQALETVQRLLPVLCQAHGLTPQQVVAIASNNGGKQALETVQRLLPVLCQAHGLTPEQVVAIASNIGGKQALETVQALLPVLCQAHGLTPEQVVAIASNIGGKQALETVQALLPVLCQAHGLTPQQVVAIASNNGGKQALETVQRLLPVLCQAHGLTPEQVVAIASNIGGKQALETVQALLPVLCQAHGLTPQQVVAIASNNGGKQALETVQRLLPVLCQAHGLTPQQVVAIASNGGGKQALETVQRLLPVLCQAHGLTPEQVVAIASNIGGKQALETVQALLPVLCQAHGLTPQQVVAIASNGGGRPALESIVAQLSRPDPSGSGSGGDPISRSQLVKSELEEKKSELRHKLKYVPHEYIELIEIARNSTQDRILEMKVMEFFMKVYGYRGKHLGGSRKPDGAIYTVGSPIDYGVIVDTKAYSGGYNLPIGQADEMQRYVEENQTRNKHINPNEWWKVYPSSVTEFKFLFVSGHFKGNYKAQLTRLNHITNCNGAVLSVEELLIGGEMIKAGTLTLEEVRRKFNNGEINFAAD

**-IL2Rα TALEN Right arm**

The aminoacid sequence of IL2Rα TALEN Right arm is documented below

MGDPKKKRKVIDYPYDVPDYAIDIADLRTLGYSQQQQEKIKPKVRSTVAQHHEALVGHGFTHAHIVALSQHPAALGTVAVKYQDMIAALPEATHEAIVGVGKQWSGARALEALLTVAGELRGPPLQLDTGQLLKIAKRGGVTAVEAVHAWRNALTGAPLNLTPQQVVAIASNNGGKQALETVQRLLPVLCQAHGLTPQQVVAIASNGGGKQALETVQRLLPVLCQAHGLTPQQVVAIASNGGGKQALETVQRLLPVLCQAHGLTPEQVVAIASHDGGKQALETVQRLLPVLCQAHGLTPQQVVAIASNGGGKQALETVQRLLPVLCQAHGLTPQQVVAIASNGGGKQALETVQRLLPVLCQAHGLTPQQVVAIASNGGGKQALETVQRLLPVLCQAHGLTPQQVVAIASNGGGKQALETVQRLLPVLCQAHGLTPQQVVAIASNNGGKQALETVQRLLPVLCQAHGLTPQQVVAIASNNGGKQALETVQRLLPVLCQAHGLTPQQVVAIASNGGGKQALETVQRLLPVLCQAHGLTPQQVVAIASNGGGKQALETVQRLLPVLCQAHGLTPQQVVAIASNGGGKQALETVQRLLPVLCQAHGLTPQQVVAIASNGGGKQALETVQRLLPVLCQAHGLTPEQVVAIASHDGGKQALETVQRLLPVLCQAHGLTPQQVVAIASNGGGRPALESIVAQLSRPDPSGSGSGGDPISRSQLVKSELEEKKSELRHKLKYVPHEYIELIEIARNSTQDRILEMKVMEFFMKVYGYRGKHLGGSRKPDGAIYTVGSPIDYGVIVDTKAYSGGYNLPIGQADEMQRYVEENQTRNKHINPNEWWKVYPSSVTEFKFLFVSGHFKGNYKAQLTRLNHITNCNGAVLSVEELLIGGEMIKAGTLTLEEVRRKFNNGEINFAAD
