# Supplementary material for: Repurposing endogenous immune pathways to tailor and control chimeric antigen receptor T cell functionality
Source: Nat Commun. 2019 Nov 13;10:5100. doi: 10.1038/s41467-019-13088-3 (PMC6853973; doi:10.1038/s41467-019-13088-3)
Supplement: Supplementary file 3 — Supplementary Data 2 [file 41467_2019_13088_MOESM3_ESM.docx]

| TRAC TALEN LEFT arm | AUGGGCGAUCCUAAAAAGAAACGUAAGGUCAUCGAUAUCGCCGAUCUACGCACGCUCGGCUACAGCCAGCAGCAACAGGAGAAGAUCAAACCGAAGGUUCGUUCGACAGUGGCGCAGCACCACGAGGCACUGGUCGGCCACGGGUUUACACACGCGCACAUCGUUGCGUUAAGCCAACACCCGGCAGCGUUAGGGACCGUCGCUGUCAAGUAUCAGGACAUGAUCGCAGCGUUGCCAGAGGCGACACACGAAGCGAUCGUUGGCGUCGGCAAACAGUGGUCCGGCGCACGCGCUCUGGAGGCCUUGCUCACGGUGGCGGGAGAGUUGAGAGGUCCACCGUUACAGUUGGACACAGGCCAACUUCUCAAGAUUGCAAAACGUGGCGGCGUGACCGCAGUGGAGGCAGUGCAUGCAUGGCGCAAUGCACUGACGGGUGCCCCGCUCAACUUGACCCCCCAGCAGGUGGUGGCCAUCGCCAGCAAUGGCGGUGGCAAGCAGGCGCUGGAGACGGUCCAGCGGCUGUUGCCGGUGCUGUGCCAGGCCCACGGCUUGACCCCCCAGCAGGUGGUGGCCAUCGCCAGCAAUAAUGGUGGCAAGCAGGCGCUGGAGACGGUCCAGCGGCUGUUGCCGGUGCUGUGCCAGGCCCACGGCUUGACCCCCCAGCAGGUGGUGGCCAUCGCCAGCAAUGGCGGUGGCAAGCAGGCGCUGGAGACGGUCCAGCGGCUGUUGCCGGUGCUGUGCCAGGCCCACGGCUUGACCCCGGAGCAGGUGGUGGCCAUCGCCAGCCACGAUGGCGGCAAGCAGGCGCUGGAGACGGUCCAGCGGCUGUUGCCGGUGCUGUGCCAGGCCCACGGCUUGACCCCGGAGCAGGUGGUGGCCAUCGCCAGCCACGAUGGCGGCAAGCAGGCGCUGGAGACGGUCCAGCGGCUGUUGCCGGUGCUGUGCCAGGCCCACGGCUUGACCCCGGAGCAGGUGGUGGCCAUCGCCAGCCACGAUGGCGGCAAGCAGGCGCUGGAGACGGUCCAGCGGCUGUUGCCGGUGCUGUGCCAGGCCCACGGCUUGACCCCGGAGCAGGUGGUGGCCAUCGCCAGCAAUAUUGGUGGCAAGCAGGCGCUGGAGACGGUGCAGGCGCUGUUGCCGGUGCUGUGCCAGGCCCACGGCUUGACCCCGGAGCAGGUGGUGGCCAUCGCCAGCCACGAUGGCGGCAAGCAGGCGCUGGAGACGGUCCAGCGGCUGUUGCCGGUGCUGUGCCAGGCCCACGGCUUGACCCCGGAGCAGGUGGUGGCCAUCGCCAGCAAUAUUGGUGGCAAGCAGGCGCUGGAGACGGUGCAGGCGCUGUUGCCGGUGCUGUGCCAGGCCCACGGCUUGACCCCCCAGCAGGUGGUGGCCAUCGCCAGCAAUAAUGGUGGCAAGCAGGCGCUGGAGACGGUCCAGCGGCUGUUGCCGGUGCUGUGCCAGGCCCACGGCUUGACCCCGGAGCAGGUGGUGGCCAUCGCCAGCAAUAUUGGUGGCAAGCAGGCGCUGGAGACGGUGCAGGCGCUGUUGCCGGUGCUGUGCCAGGCCCACGGCUUGACCCCCCAGCAGGUGGUGGCCAUCGCCAGCAAUGGCGGUGGCAAGCAGGCGCUGGAGACGGUCCAGCGGCUGUUGCCGGUGCUGUGCCAGGCCCACGGCUUGACCCCGGAGCAGGUGGUGGCCAUCGCCAGCAAUAUUGGUGGCAAGCAGGCGCUGGAGACGGUGCAGGCGCUGUUGCCGGUGCUGUGCCAGGCCCACGGCUUGACCCCCCAGCAGGUGGUGGCCAUCGCCAGCAAUGGCGGUGGCAAGCAGGCGCUGGAGACGGUCCAGCGGCUGUUGCCGGUGCUGUGCCAGGCCCACGGCUUGACCCCGGAGCAGGUGGUGGCCAUCGCCAGCCACGAUGGCGGCAAGCAGGCGCUGGAGACGGUCCAGCGGCUGUUGCCGGUGCUGUGCCAGGCCCACGGCUUGACCCCUCAGCAGGUGGUGGCCAUCGCCAGCAAUGGCGGCGGCAGGCCGGCGCUGGAGAGCAUUGUUGCCCAGUUAUCUCGCCCUGAUCCGGCGUUGGCCGCGUUGACCAACGACCACCUCGUCGCCUUGGCCUGCCUCGGCGGGCGUCCUGCGCUGGAUGCAGUGAAAAAGGGAUUGGGGGAUCCUAUCAGCCGUUCCCAGCUGGUGAAGUCCGAGCUGGAGGAGAAGAAAUCCGAGUUGAGGCACAAGCUGAAGUACGUGCCCCACGAGUACAUCGAGCUGAUCGAGAUCGCCCGGAACAGCACCCAGGACCGUAUCCUGGAGAUGAAGGUGAUGGAGUUCUUCAUGAAGGUGUACGGCUACAGGGGCAAGCACCUGGGCGGCUCCAGGAAGCCCGACGGCGCCAUCUACACCGUGGGCUCCCCCAUCGACUACGGCGUGAUCGUGGACACCAAGGCCUACUCCGGCGGCUACAACCUGCCCAUCGGCCAGGCCGACGAAAUGCAGAGGUACGUGGAGGAGAACCAGACCAGGAACAAGCACAUCAACCCCAACGAGUGGUGGAAGGUGUACCCCUCCAGCGUGACCGAGUUCAAGUUCCUGUUCGUGUCCGGCCACUUCAAGGGCAACUACAAGGCCCAGCUGACCAGGCUGAACCACAUCACCAACUGCAACGGCGCCGUGCUGUCCGUGGAGGAGCUCCUGAUCGGCGGCGAGAUGAUCAAGGCCGGCACCCUGACCCUGGAGGAGGUGAGGAGGAAGUUCAACAACGGCGAGAUCAACUUCGCGGCCGACUGAUAACUCGAGGCUGCCUUCUGCGGGGCUUGCCUUCUGGCCAUGCCCUUCUUCUCUCCCUUGCACCUGUACCUCUUGGUCUUUGAAUAAAGCCUGAGUAGGAAGGUCGAGGCGGCCAACAACAAAAAAAAAAAAAAAAAAAAAAAAAAAAAAAAAAAAAAAAAAAAAAAAAAAAAAAAAAAAAAAAAAAAAAAAAAAAAAAAAAAAAAAAAAAAAAAAAAAAAAAAAAAAAAAAAAAAAAAAA |
| --- | --- |
| TRAC TALEN RIGHT arm | AUGGGCGAUCCUAAAAAGAAACGUAAGGUCAUCGAUAUCGCCGAUCUACGCACGCUCGGCUACAGCCAGCAGCAACAGGAGAAGAUCAAACCGAAGGUUCGUUCGACAGUGGCGCAGCACCACGAGGCACUGGUCGGCCACGGGUUUACACACGCGCACAUCGUUGCGUUAAGCCAACACCCGGCAGCGUUAGGGACCGUCGCUGUCAAGUAUCAGGACAUGAUCGCAGCGUUGCCAGAGGCGACACACGAAGCGAUCGUUGGCGUCGGCAAACAGUGGUCCGGCGCACGCGCUCUGGAGGCCUUGCUCACGGUGGCGGGAGAGUUGAGAGGUCCACCGUUACAGUUGGACACAGGCCAACUUCUCAAGAUUGCAAAACGUGGCGGCGUGACCGCAGUGGAGGCAGUGCAUGCAUGGCGCAAUGCACUGACGGGUGCCCCGCUCAACUUGACCCCGGAGCAGGUGGUGGCCAUCGCCAGCCACGAUGGCGGCAAGCAGGCGCUGGAGACGGUCCAGCGGCUGUUGCCGGUGCUGUGCCAGGCCCACGGCUUGACCCCCCAGCAGGUGGUGGCCAUCGCCAGCAAUGGCGGUGGCAAGCAGGCGCUGGAGACGGUCCAGCGGCUGUUGCCGGUGCUGUGCCAGGCCCACGGCUUGACCCCGGAGCAGGUGGUGGCCAUCGCCAGCCACGAUGGCGGCAAGCAGGCGCUGGAGACGGUCCAGCGGCUGUUGCCGGUGCUGUGCCAGGCCCACGGCUUGACCCCGGAGCAGGUGGUGGCCAUCGCCAGCAAUAUUGGUGGCAAGCAGGCGCUGGAGACGGUGCAGGCGCUGUUGCCGGUGCUGUGCCAGGCCCACGGCUUGACCCCCCAGCAGGUGGUGGCCAUCGCCAGCAAUAAUGGUGGCAAGCAGGCGCUGGAGACGGUCCAGCGGCUGUUGCCGGUGCUGUGCCAGGCCCACGGCUUGACCCCGGAGCAGGUGGUGGCCAUCGCCAGCCACGAUGGCGGCAAGCAGGCGCUGGAGACGGUCCAGCGGCUGUUGCCGGUGCUGUGCCAGGCCCACGGCUUGACCCCCCAGCAGGUGGUGGCCAUCGCCAGCAAUGGCGGUGGCAAGCAGGCGCUGGAGACGGUCCAGCGGCUGUUGCCGGUGCUGUGCCAGGCCCACGGCUUGACCCCCCAGCAGGUGGUGGCCAUCGCCAGCAAUAAUGGUGGCAAGCAGGCGCUGGAGACGGUCCAGCGGCUGUUGCCGGUGCUGUGCCAGGCCCACGGCUUGACCCCCCAGCAGGUGGUGGCCAUCGCCAGCAAUAAUGGUGGCAAGCAGGCGCUGGAGACGGUCCAGCGGCUGUUGCCGGUGCUGUGCCAGGCCCACGGCUUGACCCCCCAGCAGGUGGUGGCCAUCGCCAGCAAUGGCGGUGGCAAGCAGGCGCUGGAGACGGUCCAGCGGCUGUUGCCGGUGCUGUGCCAGGCCCACGGCUUGACCCCGGAGCAGGUGGUGGCCAUCGCCAGCAAUAUUGGUGGCAAGCAGGCGCUGGAGACGGUGCAGGCGCUGUUGCCGGUGCUGUGCCAGGCCCACGGCUUGACCCCGGAGCAGGUGGUGGCCAUCGCCAGCCACGAUGGCGGCAAGCAGGCGCUGGAGACGGUCCAGCGGCUGUUGCCGGUGCUGUGCCAGGCCCACGGCUUGACCCCGGAGCAGGUGGUGGCCAUCGCCAGCAAUAUUGGUGGCAAGCAGGCGCUGGAGACGGUGCAGGCGCUGUUGCCGGUGCUGUGCCAGGCCCACGGCUUGACCCCGGAGCAGGUGGUGGCCAUCGCCAGCCACGAUGGCGGCAAGCAGGCGCUGGAGACGGUCCAGCGGCUGUUGCCGGUGCUGUGCCAGGCCCACGGCUUGACCCCCCAGCAGGUGGUGGCCAUCGCCAGCAAUAAUGGUGGCAAGCAGGCGCUGGAGACGGUCCAGCGGCUGUUGCCGGUGCUGUGCCAGGCCCACGGCUUGACCCCUCAGCAGGUGGUGGCCAUCGCCAGCAAUGGCGGCGGCAGGCCGGCGCUGGAGAGCAUUGUUGCCCAGUUAUCUCGCCCUGAUCCGGCGUUGGCCGCGUUGACCAACGACCACCUCGUCGCCUUGGCCUGCCUCGGCGGGCGUCCUGCGCUGGAUGCAGUGAAAAAGGGAUUGGGGGAUCCUAUCAGCCGUUCCCAGCUGGUGAAGUCCGAGCUGGAGGAGAAGAAAUCCGAGUUGAGGCACAAGCUGAAGUACGUGCCCCACGAGUACAUCGAGCUGAUCGAGAUCGCCCGGAACAGCACCCAGGACCGUAUCCUGGAGAUGAAGGUGAUGGAGUUCUUCAUGAAGGUGUACGGCUACAGGGGCAAGCACCUGGGCGGCUCCAGGAAGCCCGACGGCGCCAUCUACACCGUGGGCUCCCCCAUCGACUACGGCGUGAUCGUGGACACCAAGGCCUACUCCGGCGGCUACAACCUGCCCAUCGGCCAGGCCGACGAAAUGCAGAGGUACGUGGAGGAGAACCAGACCAGGAACAAGCACAUCAACCCCAACGAGUGGUGGAAGGUGUACCCCUCCAGCGUGACCGAGUUCAAGUUCCUGUUCGUGUCCGGCCACUUCAAGGGCAACUACAAGGCCCAGCUGACCAGGCUGAACCACAUCACCAACUGCAACGGCGCCGUGCUGUCCGUGGAGGAGCUCCUGAUCGGCGGCGAGAUGAUCAAGGCCGGCACCCUGACCCUGGAGGAGGUGAGGAGGAAGUUCAACAACGGCGAGAUCAACUUCGCGGCCGACUGAUAACUCGAGGCUGCCUUCUGCGGGGCUUGCCUUCUGGCCAUGCCCUUCUUCUCUCCCUUGCACCUGUACCUCUUGGUCUUUGAAUAAAGCCUGAGUAGGAAGGUCGAGGCGGCCAACAACAAAAAAAAAAAAAAAAAAAAAAAAAAAAAAAAAAAAAAAAAAAAAAAAAAAAAAAAAAAAAAAAAAAAAAAAAAAAAAAAAAAAAAAAAAAAAAAAAAAAAAAAAAAAAAAAAAAAAAAAA |
| PD1 TALEN LEFT arm | AUGGGCGAUCCUAAAAAGAAACGUAAGGUCAUCGAUUACCCAUACGAUGUUCCAGAUUACGCUAUCGAUAUCGCCGAUCUACGCACGCUCGGCUACAGCCAGCAGCAACAGGAGAAGAUCAAACCGAAGGUUCGUUCGACAGUGGCGCAGCACCACGAGGCACUGGUCGGCCACGGGUUUACACACGCGCACAUCGUUGCGUUAAGCCAACACCCGGCAGCGUUAGGGACCGUCGCUGUCAAGUAUCAGGACAUGAUCGCAGCGUUGCCAGAGGCGACACACGAAGCGAUCGUUGGCGUCGGCAAACAGUGGUCCGGCGCACGCGCUCUGGAGGCCUUGCUCACGGUGGCGGGAGAGUUGAGAGGUCCACCGUUACAGUUGGACACAGGCCAACUUCUCAAGAUUGCAAAACGUGGCGGCGUGACCGCAGUGGAGGCAGUGCAUGCAUGGCGCAAUGCACUGACGGGUGCCCCGCUCAACUUGACCCCCGAGCAAGUGGUGGCUAUCGCUUCCAAGCUGGGGGGAAAGCAGGCCCUGGAGACCGUCCAGGCCCUUCUCCCAGUGCUUUGCCAGGCUCACGGACUGACCCCUGAACAGGUGGUGGCAAUUGCCUCACACGACGGGGGCAAGCAGGCACUGGAGACUGUCCAGCGGCUGCUGCCUGUCCUCUGCCAGGCCCACGGACUCACUCCUGAGCAGGUCGUGGCCAUUGCCAGCCACGAUGGGGGCAAACAGGCUCUGGAGACCGUGCAGCGCCUCCUCCCAGUGCUGUGCCAGGCUCAUGGGCUGACCCCACAGCAGGUCGUCGCCAUUGCCAGUAACGGCGGGGGGAAGCAGGCCCUCGAAACAGUGCAGAGGCUGCUGCCCGUCUUGUGCCAAGCACACGGCCUGACACCCGAGCAGGUGGUGGCCAUCGCCUCUCAUGACGGCGGCAAGCAGGCCCUUGAGACAGUGCAGAGACUGUUGCCCGUGUUGUGUCAGGCCCACGGGUUGACACCCCAGCAGGUGGUCGCCAUCGCCAGCAAUGGCGGGGGAAAGCAGGCCCUUGAGACCGUGCAGCGGUUGCUUCCAGUGUUGUGCCAGGCACACGGACUGACCCCUCAACAGGUGGUCGCAAUCGCCAGCUACAAGGGCGGAAAGCAGGCUCUGGAGACAGUGCAGCGCCUCCUGCCCGUGCUGUGUCAGGCUCACGGACUGACACCACAGCAGGUGGUCGCCAUCGCCAGUAACGGGGGCGGCAAGCAGGCUUUGGAGACCGUCCAGAGACUCCUCCCCGUCCUUUGCCAGGCCCACGGGUUGACACCUCAGCAGGUCGUCGCCAUUGCCUCCAACAACGGGGGCAAGCAGGCCCUCGAAACUGUGCAGAGGCUGCUGCCUGUGCUGUGCCAGGCUCAUGGGCUGACACCCCAGCAGGUGGUGGCCAUUGCCUCUAACAACGGCGGCAAACAGGCACUGGAGACCGUGCAAAGGCUGCUGCCCGUCCUCUGCCAAGCCCACGGGCUCACUCCACAGCAGGUCGUGGCCAUCGCCUCAAACAAUGGCGGGAAGCAGGCCCUGGAGACUGUGCAAAGGCUGCUCCCUGUGCUCUGCCAGGCACACGGACUGACCCCUCAGCAGGUGGUGGCAAUCGCUUCCAACAACGGGGGAAAGCAGGCCCUCGAAACCGUGCAGCGCCUCCUCCCAGUGCUGUGCCAGGCACAUGGCCUCACACCCGAGCAAGUGGUGGCUAUCGCCAGCCACGACGGAGGGAAGCAGGCUCUGGAGACCGUGCAGAGGCUGCUGCCUGUCCUGUGCCAGGCCCACGGGCUUACUCCAGAGCAGGUCGUCGCCAUCGCCAGUCAUGAUGGGGGGAAGCAGGCCCUUGAGACAGUCCAGCGGCUGCUGCCAGUCCUUUGCCAGGCUCACGGCUUGACUCCCGAGCAGGUCGUGGCCAUUGCCUCAAACAUUGGGGGCAAACAGGCCCUGGAGACAGUGCAGGCCCUGCUGCCCGUGUUGUGUCAGGCCCACGGCUUGACACCCCAGCAGGUGGUCGCCAUUGCCUCUAAUGGCGGCGGGAGACCCGCCUUGGAGAGCAUUGUUGCCCAGUUAUCUCGCCCUGAUCCGGCGUUGGCCGCGUUGACCAACGACCACCUCGUCGCCUUGGCCUGCCUCGGCGGGCGUCCUGCGCUGGAUGCAGUGAAAAAGGGAUUGGGGGAUCCUAUCAGCCGUUCCCAGCUGGUGAAGUCCGAGCUGGAGGAGAAGAAAUCCGAGUUGAGGCACAAGCUGAAGUACGUGCCCCACGAGUACAUCGAGCUGAUCGAGAUCGCCCGGAACAGCACCCAGGACCGUAUCCUGGAGAUGAAGGUGAUGGAGUUCUUCAUGAAGGUGUACGGCUACAGGGGCAAGCACCUGGGCGGCUCCAGGAAGCCCGACGGCGCCAUCUACACCGUGGGCUCCCCCAUCGACUACGGCGUGAUCGUGGACACCAAGGCCUACUCCGGCGGCUACAACCUGCCCAUCGGCCAGGCCGACGAAAUGCAGAGGUACGUGGAGGAGAACCAGACCAGGAACAAGCACAUCAACCCCAACGAGUGGUGGAAGGUGUACCCCUCCAGCGUGACCGAGUUCAAGUUCCUGUUCGUGUCCGGCCACUUCAAGGGCAACUACAAGGCCCAGCUGACCAGGCUGAACCACAUCACCAACUGCAACGGCGCCGUGCUGUCCGUGGAGGAGCUCCUGAUCGGCGGCGAGAUGAUCAAGGCCGGCACCCUGACCCUGGAGGAGGUGAGGAGGAAGUUCAACAACGGCGAGAUCAACUUCGCGGCCGACUGAUAACUCGAGGCUGCCUUCUGCGGGGCUUGCCUUCUGGCCAUGCCCUUCUUCUCUCCCUUGCACCUGUACCUCUUGGUCUUUGAAUAAAGCCUGAGUAGGAAGGUCGAGGCGGCCAACAACAAAAAAAAAAAAAAAAAAAAAAAAAAAAAAAAAAAAAAAAAAAAAAAAAAAAAAAAAAAAAAAAAAAAAAAAAAAAAAAAAAAAAAAAAAAAAAAAAAAAAAAAAAAAAAAAAAAAAAAAA |
| PD1 TALEN RIGHT arm | AUGGGCGAUCCUAAAAAGAAACGUAAGGUCAUCGAUAAGGAGACCGCCGCUGCCAAGUUCGAGAGACAGCACAUGGACAGCAUCGAUAUCGCCGAUCUACGCACGCUCGGCUACAGCCAGCAGCAACAGGAGAAGAUCAAACCGAAGGUUCGUUCGACAGUGGCGCAGCACCACGAGGCACUGGUCGGCCACGGGUUUACACACGCGCACAUCGUUGCGUUAAGCCAACACCCGGCAGCGUUAGGGACCGUCGCUGUCAAGUAUCAGGACAUGAUCGCAGCGUUGCCAGAGGCGACACACGAAGCGAUCGUUGGCGUCGGCAAACAGUGGUCCGGCGCACGCGCUCUGGAGGCCUUGCUCACGGUGGCGGGAGAGUUGAGAGGUCCACCGUUACAGUUGGACACAGGCCAACUUCUCAAGAUUGCAAAACGUGGCGGCGUGACCGCAGUGGAGGCAGUGCAUGCAUGGCGCAAUGCACUGACGGGUGCCCCGCUCAACUUGACCCCCGAGCAAGUCGUCGCAAUCGCCAGCCAUGAUGGAGGGAAGCAAGCCCUCGAAACCGUGCAGCGGUUGCUUCCUGUGCUCUGCCAGGCCCACGGCCUUACCCCUCAGCAGGUGGUGGCCAUCGCAAGUAACGGAGGAGGAAAGCAAGCCUUGGAGACAGUGCAGCGCCUGUUGCCCGUGCUGUGCCAGGCACACGGCCUCACACCAGAGCAGGUCGUGGCCAUUGCCUCCCAUGACGGGGGGAAACAGGCUCUGGAGACCGUCCAGAGGCUGCUGCCCGUCCUCUGUCAAGCUCACGGCCUGACUCCCCAACAAGUGGUCGCCAUCGCCUCUAAUGGCGGCGGGAAGCAGGCACUGGAAACAGUGCAGAGACUGCUCCCUGUGCUUUGCCAAGCUCAUGGGUUGACCCCCCAACAGGUCGUCGCUAUUGCCUCAAACGGGGGGGGCAAGCAGGCCCUUGAGACUGUGCAGAGGCUGUUGCCAGUGCUGUGUCAGGCUCACGGGCUCACUCCACAACAGGUGGUCGCAAUUGCCAGCAACGGCGGCGGAAAGCAAGCUCUUGAAACCGUGCAACGCCUCCUGCCCGUGCUCUGUCAGGCUCAUGGCCUGACACCACAACAAGUCGUGGCCAUCGCCAGUAAUAAUGGCGGGAAACAGGCUCUUGAGACCGUCCAGAGGCUGCUCCCAGUGCUCUGCCAGGCACACGGGCUGACCCCCGAGCAGGUGGUGGCUAUCGCCAGCAAUAUUGGGGGCAAGCAGGCCCUGGAAACAGUCCAGGCCCUGCUGCCAGUGCUUUGCCAGGCUCACGGGCUCACUCCCCAGCAGGUCGUGGCAAUCGCCUCCAACGGCGGAGGGAAGCAGGCUCUGGAGACCGUGCAGAGACUGCUGCCCGUCUUGUGCCAGGCCCACGGACUCACACCUGAACAGGUCGUCGCCAUUGCCUCUCACGAUGGGGGCAAACAAGCCCUGGAGACAGUGCAGCGGCUGUUGCCUGUGUUGUGCCAAGCCCACGGCUUGACUCCUCAACAAGUGGUCGCCAUCGCCUCAAAUGGCGGCGGAAAACAAGCUCUGGAGACAGUGCAGAGGUUGCUGCCCGUCCUCUGCCAAGCCCACGGCCUGACUCCCCAACAGGUCGUCGCCAUUGCCAGCAACAACGGAGGAAAGCAGGCUCUCGAAACUGUGCAGCGGCUGCUUCCUGUGCUGUGUCAGGCUCAUGGGCUGACCCCCGAGCAAGUGGUGGCUAUUGCCUCUAAUGGAGGCAAGCAAGCCCUUGAGACAGUCCAGAGGCUGUUGCCAGUGCUGUGCCAGGCCCACGGGCUCACACCCCAGCAGGUGGUCGCCAUCGCCAGUAACAACGGGGGCAAACAGGCAUUGGAAACCGUCCAGCGCCUGCUUCCAGUGCUCUGCCAGGCACACGGACUGACACCCGAACAGGUGGUGGCCAUUGCAUCCCAUGAUGGGGGCAAGCAGGCCCUGGAGACCGUGCAGAGACUCCUGCCAGUGUUGUGCCAAGCUCACGGCCUCACCCCUCAGCAAGUCGUGGCCAUCGCCUCAAACGGGGGGGGCCGGCCUGCACUGGAGAGCAUUGUUGCCCAGUUAUCUCGCCCUGAUCCGGCGUUGGCCGCGUUGACCAACGACCACCUCGUCGCCUUGGCCUGCCUCGGCGGGCGUCCUGCGCUGGAUGCAGUGAAAAAGGGAUUGGGGGAUCCUAUCAGCCGUUCCCAGCUGGUGAAGUCCGAGCUGGAGGAGAAGAAAUCCGAGUUGAGGCACAAGCUGAAGUACGUGCCCCACGAGUACAUCGAGCUGAUCGAGAUCGCCCGGAACAGCACCCAGGACCGUAUCCUGGAGAUGAAGGUGAUGGAGUUCUUCAUGAAGGUGUACGGCUACAGGGGCAAGCACCUGGGCGGCUCCAGGAAGCCCGACGGCGCCAUCUACACCGUGGGCUCCCCCAUCGACUACGGCGUGAUCGUGGACACCAAGGCCUACUCCGGCGGCUACAACCUGCCCAUCGGCCAGGCCGACGAAAUGCAGAGGUACGUGGAGGAGAACCAGACCAGGAACAAGCACAUCAACCCCAACGAGUGGUGGAAGGUGUACCCCUCCAGCGUGACCGAGUUCAAGUUCCUGUUCGUGUCCGGCCACUUCAAGGGCAACUACAAGGCCCAGCUGACCAGGCUGAACCACAUCACCAACUGCAACGGCGCCGUGCUGUCCGUGGAGGAGCUCCUGAUCGGCGGCGAGAUGAUCAAGGCCGGCACCCUGACCCUGGAGGAGGUGAGGAGGAAGUUCAACAACGGCGAGAUCAACUUCGCGGCCGACUGAUAACUCGAGGCUGCCUUCUGCGGGGCUUGCCUUCUGGCCAUGCCCUUCUUCUCUCCCUUGCACCUGUACCUCUUGGUCUUUGAAUAAAGCCUGAGUAGGAAGGUCGAGGCGGCCAACAACAAAAAAAAAAAAAAAAAAAAAAAAAAAAAAAAAAAAAAAAAAAAAAAAAAAAAAAAAAAAAAAAAAAAAAAAAAAAAAAAAAAAAAAAAAAAAAAAAAAAAAAAAAAAAAAAAAAAAAAAA |
| IL2Rα TALEN LEFT arm | AUGGGCGAUCCUAAAAAGAAACGUAAGGUCAUCGAUUACCCAUACGAUGUUCCAGAUUACGCUAUCGAUAUCGCCGAUCUACGCACGCUCGGCUACAGCCAGCAGCAACAGGAGAAGAUCAAACCGAAGGUUCGUUCGACAGUGGCGCAGCACCACGAGGCACUGGUCGGCCACGGGUUUACACACGCGCACAUCGUUGCGUUAAGCCAACACCCGGCAGCGUUAGGGACCGUCGCUGUCAAGUAUCAGGACAUGAUCGCAGCGUUGCCAGAGGCGACACACGAAGCGAUCGUUGGCGUCGGCAAACAGUGGUCCGGCGCACGCGCUCUGGAGGCCUUGCUCACGGUGGCGGGAGAGUUGAGAGGUCCACCGUUACAGUUGGACACAGGCCAACUUCUCAAGAUUGCAAAACGUGGCGGCGUGACCGCAGUGGAGGCAGUGCAUGCAUGGCGCAAUGCACUGACGGGUGCCCCGCUCAACUUGACCCCGGAGCAGGUGGUGGCCAUCGCCAGCAAUAUUGGUGGCAAGCAGGCGCUGGAGACGGUGCAGGCGCUGUUGCCGGUGCUGUGCCAGGCCCACGGCUUGACCCCGGAGCAGGUGGUGGCCAUCGCCAGCCACGAUGGCGGCAAGCAGGCGCUGGAGACGGUCCAGCGGCUGUUGCCGGUGCUGUGCCAGGCCCACGGCUUGACCCCGGAGCAGGUGGUGGCCAUCGCCAGCAAUAUUGGUGGCAAGCAGGCGCUGGAGACGGUGCAGGCGCUGUUGCCGGUGCUGUGCCAGGCCCACGGCUUGACCCCCCAGCAGGUGGUGGCCAUCGCCAGCAAUAAUGGUGGCAAGCAGGCGCUGGAGACGGUCCAGCGGCUGUUGCCGGUGCUGUGCCAGGCCCACGGCUUGACCCCCCAGCAGGUGGUGGCCAUCGCCAGCAAUAAUGGUGGCAAGCAGGCGCUGGAGACGGUCCAGCGGCUGUUGCCGGUGCUGUGCCAGGCCCACGGCUUGACCCCGGAGCAGGUGGUGGCCAUCGCCAGCAAUAUUGGUGGCAAGCAGGCGCUGGAGACGGUGCAGGCGCUGUUGCCGGUGCUGUGCCAGGCCCACGGCUUGACCCCCCAGCAGGUGGUGGCCAUCGCCAGCAAUAAUGGUGGCAAGCAGGCGCUGGAGACGGUCCAGCGGCUGUUGCCGGUGCUGUGCCAGGCCCACGGCUUGACCCCCCAGCAGGUGGUGGCCAUCGCCAGCAAUAAUGGUGGCAAGCAGGCGCUGGAGACGGUCCAGCGGCUGUUGCCGGUGCUGUGCCAGGCCCACGGCUUGACCCCGGAGCAGGUGGUGGCCAUCGCCAGCAAUAUUGGUGGCAAGCAGGCGCUGGAGACGGUGCAGGCGCUGUUGCCGGUGCUGUGCCAGGCCCACGGCUUGACCCCGGAGCAGGUGGUGGCCAUCGCCAGCAAUAUUGGUGGCAAGCAGGCGCUGGAGACGGUGCAGGCGCUGUUGCCGGUGCUGUGCCAGGCCCACGGCUUGACCCCCCAGCAGGUGGUGGCCAUCGCCAGCAAUAAUGGUGGCAAGCAGGCGCUGGAGACGGUCCAGCGGCUGUUGCCGGUGCUGUGCCAGGCCCACGGCUUGACCCCGGAGCAGGUGGUGGCCAUCGCCAGCAAUAUUGGUGGCAAGCAGGCGCUGGAGACGGUGCAGGCGCUGUUGCCGGUGCUGUGCCAGGCCCACGGCUUGACCCCCCAGCAGGUGGUGGCCAUCGCCAGCAAUAAUGGUGGCAAGCAGGCGCUGGAGACGGUCCAGCGGCUGUUGCCGGUGCUGUGCCAGGCCCACGGCUUGACCCCCCAGCAGGUGGUGGCCAUCGCCAGCAAUGGCGGUGGCAAGCAGGCGCUGGAGACGGUCCAGCGGCUGUUGCCGGUGCUGUGCCAGGCCCACGGCUUGACCCCGGAGCAGGUGGUGGCCAUCGCCAGCAAUAUUGGUGGCAAGCAGGCGCUGGAGACGGUGCAGGCGCUGUUGCCGGUGCUGUGCCAGGCCCACGGCUUGACCCCUCAGCAGGUGGUGGCCAUCGCCAGCAAUGGCGGCGGCAGGCCGGCGCUGGAGAGCAUUGUUGCCCAGUUAUCUCGCCCUGAUCCGAGUGGCAGCGGAAGUGGCGGGGAUCCUAUCAGCCGUUCCCAGCUGGUGAAGUCCGAGCUGGAGGAGAAGAAAUCCGAGUUGAGGCACAAGCUGAAGUACGUGCCCCACGAGUACAUCGAGCUGAUCGAGAUCGCCCGGAACAGCACCCAGGACCGUAUCCUGGAGAUGAAGGUGAUGGAGUUCUUCAUGAAGGUGUACGGCUACAGGGGCAAGCACCUGGGCGGCUCCAGGAAGCCCGACGGCGCCAUCUACACCGUGGGCUCCCCCAUCGACUACGGCGUGAUCGUGGACACCAAGGCCUACUCCGGCGGCUACAACCUGCCCAUCGGCCAGGCCGACGAAAUGCAGAGGUACGUGGAGGAGAACCAGACCAGGAACAAGCACAUCAACCCCAACGAGUGGUGGAAGGUGUACCCCUCCAGCGUGACCGAGUUCAAGUUCCUGUUCGUGUCCGGCCACUUCAAGGGCAACUACAAGGCCCAGCUGACCAGGCUGAACCACAUCACCAACUGCAACGGCGCCGUGCUGUCCGUGGAGGAGCUCCUGAUCGGCGGCGAGAUGAUCAAGGCCGGCACCCUGACCCUGGAGGAGGUGAGGAGGAAGUUCAACAACGGCGAGAUCAACUUCGCGGCCGACUGAUAACUCGAGGCUGCCUUCUGCGGGGCUUGCCUUCUGGCCAUGCCCUUCUUCUCUCCCUUGCACCUGUACCUCUUGGUCUUUGAAUAAAGCCUGAGUAGGAAGGUCGAGGCGGCCAACAACAAAAAAAAAAAAAAAAAAAAAAAAAAAAAAAAAAAAAAAAAAAAAAAAAAAAAAAAAAAAAAAAAAAAAAAAAAAAAAAAAAAAAAAAAAAAAAAAAAAAAAAAAAAAAAAAAAAAAAAAA |
| IL2Rα TALEN RIGHT arm | AUGGGCGAUCCUAAAAAGAAACGUAAGGUCAUCGAUUACCCAUACGAUGUUCCAGAUUACGCUAUCGAUAUCGCCGAUCUACGCACGCUCGGCUACAGCCAGCAGCAACAGGAGAAGAUCAAACCGAAGGUUCGUUCGACAGUGGCGCAGCACCACGAGGCACUGGUCGGCCACGGGUUUACACACGCGCACAUCGUUGCGUUAAGCCAACACCCGGCAGCGUUAGGGACCGUCGCUGUCAAGUAUCAGGACAUGAUCGCAGCGUUGCCAGAGGCGACACACGAAGCGAUCGUUGGCGUCGGCAAACAGUGGUCCGGCGCACGCGCUCUGGAGGCCUUGCUCACGGUGGCGGGAGAGUUGAGAGGUCCACCGUUACAGUUGGACACAGGCCAACUUCUCAAGAUUGCAAAACGUGGCGGCGUGACCGCAGUGGAGGCAGUGCAUGCAUGGCGCAAUGCACUGACGGGUGCCCCGCUCAACUUGACCCCCCAGCAGGUGGUGGCCAUCGCCAGCAAUAAUGGUGGCAAGCAGGCGCUGGAGACGGUCCAGCGGCUGUUGCCGGUGCUGUGCCAGGCCCACGGCUUGACCCCCCAGCAGGUGGUGGCCAUCGCCAGCAAUGGCGGUGGCAAGCAGGCGCUGGAGACGGUCCAGCGGCUGUUGCCGGUGCUGUGCCAGGCCCACGGCUUGACCCCCCAGCAGGUGGUGGCCAUCGCCAGCAAUGGCGGUGGCAAGCAGGCGCUGGAGACGGUCCAGCGGCUGUUGCCGGUGCUGUGCCAGGCCCACGGCUUGACCCCGGAGCAGGUGGUGGCCAUCGCCAGCCACGAUGGCGGCAAGCAGGCGCUGGAGACGGUCCAGCGGCUGUUGCCGGUGCUGUGCCAGGCCCACGGCUUGACCCCCCAGCAGGUGGUGGCCAUCGCCAGCAAUGGCGGUGGCAAGCAGGCGCUGGAGACGGUCCAGCGGCUGUUGCCGGUGCUGUGCCAGGCCCACGGCUUGACCCCCCAGCAGGUGGUGGCCAUCGCCAGCAAUGGCGGUGGCAAGCAGGCGCUGGAGACGGUCCAGCGGCUGUUGCCGGUGCUGUGCCAGGCCCACGGCUUGACCCCCCAGCAGGUGGUGGCCAUCGCCAGCAAUGGCGGUGGCAAGCAGGCGCUGGAGACGGUCCAGCGGCUGUUGCCGGUGCUGUGCCAGGCCCACGGCUUGACCCCCCAGCAGGUGGUGGCCAUCGCCAGCAAUGGCGGUGGCAAGCAGGCGCUGGAGACGGUCCAGCGGCUGUUGCCGGUGCUGUGCCAGGCCCACGGCUUGACCCCCCAGCAGGUGGUGGCCAUCGCCAGCAAUAAUGGUGGCAAGCAGGCGCUGGAGACGGUCCAGCGGCUGUUGCCGGUGCUGUGCCAGGCCCACGGCUUGACCCCCCAGCAGGUGGUGGCCAUCGCCAGCAAUAAUGGUGGCAAGCAGGCGCUGGAGACGGUCCAGCGGCUGUUGCCGGUGCUGUGCCAGGCCCACGGCUUGACCCCCCAGCAGGUGGUGGCCAUCGCCAGCAAUGGCGGUGGCAAGCAGGCGCUGGAGACGGUCCAGCGGCUGUUGCCGGUGCUGUGCCAGGCCCACGGCUUGACCCCCCAGCAGGUGGUGGCCAUCGCCAGCAAUGGCGGUGGCAAGCAGGCGCUGGAGACGGUCCAGCGGCUGUUGCCGGUGCUGUGCCAGGCCCACGGCUUGACCCCCCAGCAGGUGGUGGCCAUCGCCAGCAAUGGCGGUGGCAAGCAGGCGCUGGAGACGGUCCAGCGGCUGUUGCCGGUGCUGUGCCAGGCCCACGGCUUGACCCCCCAGCAGGUGGUGGCCAUCGCCAGCAAUGGCGGUGGCAAGCAGGCGCUGGAGACGGUCCAGCGGCUGUUGCCGGUGCUGUGCCAGGCCCACGGCUUGACCCCGGAGCAGGUGGUGGCCAUCGCCAGCCACGAUGGCGGCAAGCAGGCGCUGGAGACGGUCCAGCGGCUGUUGCCGGUGCUGUGCCAGGCCCACGGCUUGACCCCUCAGCAGGUGGUGGCCAUCGCCAGCAAUGGCGGCGGCAGGCCGGCGCUGGAGAGCAUUGUUGCCCAGUUAUCUCGCCCUGAUCCGAGUGGCAGCGGAAGUGGCGGGGAUCCUAUCAGCCGUUCCCAGCUGGUGAAGUCCGAGCUGGAGGAGAAGAAAUCCGAGUUGAGGCACAAGCUGAAGUACGUGCCCCACGAGUACAUCGAGCUGAUCGAGAUCGCCCGGAACAGCACCCAGGACCGUAUCCUGGAGAUGAAGGUGAUGGAGUUCUUCAUGAAGGUGUACGGCUACAGGGGCAAGCACCUGGGCGGCUCCAGGAAGCCCGACGGCGCCAUCUACACCGUGGGCUCCCCCAUCGACUACGGCGUGAUCGUGGACACCAAGGCCUACUCCGGCGGCUACAACCUGCCCAUCGGCCAGGCCGACGAAAUGCAGAGGUACGUGGAGGAGAACCAGACCAGGAACAAGCACAUCAACCCCAACGAGUGGUGGAAGGUGUACCCCUCCAGCGUGACCGAGUUCAAGUUCCUGUUCGUGUCCGGCCACUUCAAGGGCAACUACAAGGCCCAGCUGACCAGGCUGAACCACAUCACCAACUGCAACGGCGCCGUGCUGUCCGUGGAGGAGCUCCUGAUCGGCGGCGAGAUGAUCAAGGCCGGCACCCUGACCCUGGAGGAGGUGAGGAGGAAGUUCAACAACGGCGAGAUCAACUUCGCGGCCGACUGAUAACUCGAGGCUGCCUUCUGCGGGGCUUGCCUUCUGGCCAUGCCCUUCUUCUCUCCCUUGCACCUGUACCUCUUGGUCUUUGAAUAAAGCCUGAGUAGGAAGGUCGAGGCGGCCAACAACAAAAAAAAAAAAAAAAAAAAAAAAAAAAAAAAAAAAAAAAAAAAAAAAAAAAAAAAAAAAAAAAAAAAAAAAAAAAAAAAAAAAAAAAAAAAAAAAAAAAAAAAAAAAAAAAAAAAAAAAA |
